# Genomic Analysis and Virulence Features of *Vibrio cholerae* Non‐O1/Non‐O139 Harbouring CARB‐Type β‐Lactamases From Freshwater Bodies, Argentina

**DOI:** 10.1111/1758-2229.70181

**Published:** 2025-09-25

**Authors:** Daiana Guevara Núñez, Fabrizzio N. Morandini, Geehan Suleyman, Kyle Crooker, Jagjeet Kaur, Gina Maki, José L. Bocco, Darío Fernández Do Porto, Markus J. Zervos, Claudia Sola, H. Alex Saka

**Affiliations:** ^1^ Instituto de Química, Física de los Materiales, Medioambiente y Energía (IQUIBICEN), Consejo Nacional de Investigaciones Científicas y Técnicas (CONICET), Facultad de Ciencias Exactas y Naturales, Universidad Nacional de Buenos Aires Ciudad Autónoma de Buenos Aires Argentina; ^2^ Centro de Investigaciones en Bioquímica Clínica e Inmunología (CIBICI), Consejo Nacional de Investigaciones Científicas y Técnicas (CONICET), Departamento de Bioquímica Clínica, Facultad de Ciencias Químicas, Universidad Nacional de Córdoba Córdoba Provincia de Córdoba Argentina; ^3^ Henry Ford Health System Detroit Michigan USA; ^4^ Global Health Initiative Henry Ford Health System Detroit Michigan USA; ^5^ Wayne State University Detroit Michigan USA

**Keywords:** antibiotic resistance, bacteria, beta‐lactamases, infectious agents in the environment

## Abstract

*Vibrio cholerae* is a globally distributed, free‐living bacterium in aquatic ecosystems. While non‐O1/non‐O139 serogroups typically do not produce cholera toxin, they have the potential to cause diarrhoea. These strains may act as reservoirs of antibiotic resistance in rivers, lakes and oceans. Understanding their genetic resistance and virulence can shed light on their role in spreading antimicrobial resistance and their pathogenicity. In this study, we characterised 60 *V. cholerae* non‐O1/non‐O139 strains from 16 freshwater bodies located throughout the Province of Córdoba, Argentina. We found none of the strains carried cholera toxin and identified ampicillin resistance as the most prevalent phenotype. Whole genome sequencing revealed that all ampicillin‐resistant strains (*n* = 10) carried CARB β‐lactamases, leading to the identification of new CARB variants (CARB‐59 to CARB‐62) likely associated with the *V. cholerae* superintegron. Two strains were notably related and exhibited enhanced virulence due to an unusual genetic arrangement of the VPI‐1 pathogenicity island, encoding both the toxin co‐regulated pilus and a type VI secretion system cluster subclass i5, commonly found in non‐cholera *Vibrio* species. These findings provide significant insights into the genetic diversity and virulent potential of ampicillin‐resistant environmental *V. cholerae* non‐O1/non‐O139 and enhance our understanding of the evolution of CARB β‐lactamases within the species.

## Graphical abstract

Genomic analysis of ampicillin‐resistant *Vibrio cholerae* non‐O1/non‐O139 from central Argentina's freshwater bodies identified four novel CARB‐β‐lactamase variants linked to the *V. cholerae* superintegron. An unusual VPI‐1 arrangement encoding the TCP pilus and a type VI secretion system subclass i5 was also found, associated with increased enterotoxigenicity. Our findings highlight *V. cholerae* as a reservoir of resistance and virulence genes and support that CARB‐β‐lactamases play important roles in the microbial interactions of these bacteria in the environment independently of strong anthropogenic antibiotic pressures.

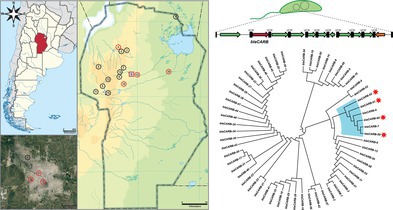

## 1 Introduction

*Vibrio cholerae* is a Gram‐negative, comma‐shaped bacterium that is widely distributed in aquatic ecosystems around the globe. This bacterium is subclassified into more than 200 serogroups based on differences in the O‐antigen of the lipopolysaccharide. Importantly, *V. cholerae* serogroups O1 and O139 are the causative agents of cholera pandemics, primarily due to the expression of major virulence factors: cholera toxin (CT) and toxin co‐regulated pilus (TCP) (Montero et al. 2023). CT, a potent enterotoxin, causes the characteristic explosive watery diarrhoea of cholera syndrome. Cholera, which is transmitted through person‐to‐person contact or via ingestion of contaminated food or water, mainly in developing countries, was estimated to have caused 1.3–4.0 million cases and 21,000 to 143,000 deaths per year globally (WHO 2024). Remarkably, CT is encoded by the *ctxAB* genes within a filamentous bacteriophage CTXϕ, which also carries the accessory cholera enterotoxin (*ace*) and the zonula occludens toxin (*zot*) (Waldor and Mekalanos 1996). TCP, a type IV pilus encoded in the *V. cholerae* pathogenicity island VPI‐1, serves dual roles: as the natural receptor for CTXϕ and a key colonisation factor mediating adherence to intestinal epithelial cells (Kumar et al. 2020). Other serogroups, typically lacking CT and TCP, are collectively known as *V. cholerae* non‐O1/non‐O139. While they do not have epidemic or pandemic potential, these strains can cause sporadic cases and limited outbreaks of mild to moderate diarrhoea or even cholera‐like symptoms (Montero et al. 2023; Dutta et al. 2013; Arteaga et al. 2020). Most *V. cholerae* non‐O1/non‐O139 strains express a capsule linked to their ability to cause various extraintestinal diseases, including soft tissue infections, ear infections and fatal cases of bacteremia and sepsis (Blake et al. 1980; Chowdhury et al. 2016; Maraki et al. 2016; Chen et al. 2007). In recent years, the pathogenic role of *V. cholerae* non‐O1/non‐O139 strains has gained attention due to rising trends associated with global climate change (Vezzulli et al. 2020). Additionally, they are considered foodborne pathogens associated with the consumption of raw or undercooked seafood (Zhang et al. 2024).

Antimicrobial resistance stands as one of the foremost challenges to human health today. Recent studies estimate that 1.14 million deaths were attributable to antimicrobial resistance in 2021, with a staggering forecast of 39.1 million deaths projected between 2025 and 2050 (Collaborators GBDAR 2024). This worrying scenario requires a comprehensive approach to strengthening our understanding of the underlying causes of antimicrobial resistance. There is a growing consensus that antimicrobial resistance should be viewed as a multifactorial problem, consisting of intricate interactions among humans, animals, microorganisms, and their environment. This perspective gave rise to the ‘One Health’ approach to antimicrobial resistance (Collignon et al. 2018; Laxminarayan et al. 2013; White and Hughes 2019; McEwen and Collignon 2018). In this context, significant gaps remain in our understanding of environmental antimicrobial resistance, particularly regarding resistances associated with free‐living bacteria in aquatic ecosystems (Sharda et al. 2023; Skandalis et al. 2021). *V. cholerae* , a bacterium highly adapted to various water bodies, could serve as a significant reservoir of antibiotic resistance genes in the environment (Ceccarelli et al. 2016). Furthermore, the genomic plasticity of this microorganism facilitates genetic exchange via mobile elements such as plasmids, integrative conjugative elements (ICE), class I integrons and the *V. cholerae* superintegron (Carraro et al. 2016; Waldor et al. 1996; Hochhut et al. 2001; Zuberi and Sillo 2022; Mazel et al. 1998).

In this study, we conducted a detailed characterisation of environmental *V. cholerae* non‐O1/non‐O139 strains isolated from diverse bodies of water in Córdoba Province, at the geographic centre of Argentina. Interestingly, we identified ampicillin resistance as the most prevalent phenotype, consistent with previous reports from freshwater environments in other regions of Argentina (Fraga et al. 2007). Although several studies have used whole‐genome sequencing (WGS) to analyse antimicrobial resistance in environmental *V. cholerae* non‐O1/non‐O139 strains from various countries (Bhandari et al. 2023; Schmidt et al. 2023; Lepuschitz et al. 2019; Jia et al. 2025; Siriphap et al. 2017), no such analysis has been reported in Argentina. Thus, we utilised WGS to carry out a detailed molecular characterisation of ampicillin‐resistant *V. cholerae* non‐O1/non‐O139 strains identified in this study. Our findings revealed that ampicillin resistance in all strains was attributed to CARB family β‐lactamases. This analysis led to the discovery of four previously unreported CARB variants (CARB‐59 to CARB‐62), likely associated with the *V. cholerae* superintegron. Furthermore, we elucidated their virulome, assessed their enterotoxigenicity and conducted phylogenetic analyses to determine the genetic relationships between the strains. Notably, we found that two strains were highly related and exhibited increased virulence, which was linked to an unusual genetic arrangement of the VPI‐1 island containing a type VI secretion system (TVISS) large cluster.

## 2 Experimental Procedures

### 2.1 Bacterial Strains

A total of 60 *V. cholerae* non‐O1/non‐O139 strains from environmental freshwater sources in the province of Córdoba, Argentina, obtained from 1991 to 1994 in the context of a cholera surveillance program and cryopreserved at −80°C in the CIBICI‐CONICET *V. cholerae* collection, were included in this work. The isolation sites and dates for these strains are detailed in Table S1. In addition, 2 CT‐negative *V. cholerae* non‐O1/non‐O139 and 1 cholera‐toxin positive *V. cholerae* O1 El Tor clinical isolate (VC4, VC44 and EP1/151, respectively, CIBICI‐CONICET *V. cholerae* collection), as well as *Escherichia coli* DH5α were included for selected assays, as specified. All *V. cholerae* strains were identified by classical culture and microbiological methods and agglutination with O1 and O139 antisera.

### 2.2 Antimicrobial Susceptibility Testing

Antimicrobial susceptibility profiles to all environmental *V. cholerae* non‐O1/non‐O139 strains were determined by the disk diffusion method according to the recommendations and interpretive criteria of the Clinical and Laboratory Standards Institute (CLSI) for *Vibrio* spp. (including *V. cholerae* ) (CLSI 2015) to the following antibiotics: ampicillin (10 μg), gentamicin (10 μg), tetracycline (30 μg), ciprofloxacin (5 μg), trimethoprim/sulfamethoxazole (1.25/23.75 μg) and chloramphenicol (30 μg). For gentamicin and ciprofloxacin, breakpoints applicable for *Vibrio* spp. other than *V. cholerae* were considered. In addition, susceptibility to cefazolin (30 μg) was tested by disk diffusion according to CLSI recommendations and interpretive criteria for *Enterobacterales* (excluding *Salmonella* and *Shigella* spp.) (CLSI 2024a). All ampicillin‐resistant strains as well as a subset of ampicillin‐susceptible strains were further tested by agar dilution method for determination of the MICs to ampicillin, ampicillin/sulbactam, amoxicillin/clavulanic acid, ticarcillin, ticarcillin/clavulanic acid, piperacillin, cefazolin, cefoxitin and cefotaxime, following CLSI recommendations (CLSI 2024b).

### 2.3 Isoelectric Focusing

Analytical isoelectric focusing was performed as previously described (Saka et al. 2003) with minor modifications. Briefly, single colonies of each ampicillin‐resistant strains (*n* = 10) were grown in 10 mL of brain heart infusion broth overnight at 37°C in shaking conditions (120 rpm). Overnight cultures were centrifuged (20 min, 5000 × *g*) at room temperature, bacterial pellets were resuspended on phosphate‐buffered saline (PBS) 0.01M (pH = 7), transferred to Eppendorf tubes and subjected to freeze–thaw cycles as previously described for obtaining crude β‐lactamase extracts (Livermore and Williams 1996). Isoelectric focusing was carried out on pH: 3.0–10.0 (Bio‐Lyte 3/10 Ampholyte, BioRad) polyacrylamide gels as described elsewhere (Sanders et al. 1986). The β‐lactamase bands were visualised by the iodometric method (Labia and Barthelemy 1979), employing ampicillin (100 μg/mL) as developing substrates. β‐lactamases of known isoelectric points (pI) kindly provided by Servicio Antimicrobianos, ‘INEI‐ANLIS Dr. C. G. Malbrán’ were used as standards: PER‐2 (pI 5.4, *E. coli* strain M1857) and CTX‐M‐2 (pI 7.9, *E. coli* strain M1890). Predicted isolelectric points were obtained using the bioinformatic tool IPC—Isoelectric Point Calculator (Kozlowski 2016) available at http://isoelectric.ovh.org.

### 2.4 Polymerase Chain Reaction (PCR) Detection of Virulence Genes

PCR reactions were used for detection of the following virulence genes: *hlyA* ( *V. cholerae* cytolysin) (Saka et al. 2008), *toxR* (transcriptional activator ToxR/CadC) (Parsot and Mekalanos 1990), *ctxA* (CT A subunit) (Mekalanos et al. 1983), *tcpA* (toxin‐coregulated pilus major structural subunit), *ace* (accessory cholera enterotoxin) (Waldor and Mekalanos 1996) and *zot* (zonula occludens toxin). Primer sequences are shown in Table S2. PCR conditions were carried out as previously described (Bidinost et al. 2004).

### 2.5 Deoxyribonucleic Acid (DNA) Extraction and Whole Genome Sequencing (WGS)

All the ampicillin‐resistant strains (*n* = 10) were selected for WGS. Genomic DNA from axenic overnight cultures was extracted using the commercial DNeasy Blood & Tissue extraction kit (QIAGEN) following the manufacturer's instructions and then sequenced at the Genome Sciences Core Laboratory, Wayne State University, Detroit, Michigan, USA on the Illumina Novaseq 6000 platform with 150 bp paired‐end reads.

### 2.6 Reads and De Novo Genome Assembly Quality Controls

Sequence reads were trimmed with Trimmomatic v0.39 (Bolger et al. 2014) and quality was assessed by FastQC v0.11.9 (Andrews 2010). De novo assemblies were generated with SPAdes 3.15.2 (Bankevich et al. 2012), and the quality of the assemblies was evaluated with QUAST 5.0.2 (Gurevich et al. 2013) (Table S3). The coverage, expressed as the mean sequencing depth per contig, ranged from 122× to 340×, and genome gaps, expressed as ‘#N's per 100 Kbp’, ranged from 2.35 to 17.57, as detailed in Table S3. Genome purity checks were conducted using CheckM (Parks et al. 2015) (Table S4). WGS assemblies of each strain were deposited in NCBI database with the following accession numbers: JBFNBT000000000 (VC3), JBFNBU000000000 (VC12), JBFNBV000000000 (VC36), JBFNBW000000000 (VC41), JBFNBX000000000 (VC58), JBFNBY000000000 (VC77), JBFNBZ000000000 (VC84), JBFNCA000000000 (VC92), JBFNCB000000000 (VC95), JBFNCC000000000 (VC97).

### 2.7 Multilocus Sequence Typing

To confirm bacterial species, whole genome assemblies of all ampicillin‐resistant strains (*n* = 10) were analysed by Ribosomal Multilocus Sequence Typing (rMLST) web server (Jolley et al. 2012) available at https://pubmlst.org/species‐id. Multilocus Sequence Typing (MLST) was carried out using the pubMLST bioinformatic tool (Jolley et al. 2018) available at https://pubmlst.org/organisms/vibrio‐cholerae. The *V. cholerae* non‐O1/non‐O139 MLST scheme, described in (Octavia et al. 2013), is based on the following housekeeping genes: *adk* (adenylate kinase), *gyrB* (DNA gyrase subunit B), *mdh* (malate dehydrogenase), *metE* (methionine synthase), *pntA* (pyiridine nucleotide transhydrogenase), *purM* (phosphoribosyl‐formylglycinamide cyclo‐ligase) and *pyrC* (dihydroorotase).

### 2.8 Resistome, Virulome and Plasmid Analysis

Antibiotic resistance genes of all ampicillin‐resistant strains (*n* = 10), including *bla*_*CARB*_ alleles, were identified using AMRFinderPlus (Feldgarden et al. 2021) on the de novo assemblies, as well as ARIBA (Hunt et al. 2017) on the trimmed reads. All positive resistance genes complied with a cut‐off value of 90% coverage and 95% nucleotide identity. In addition, virulence genes of these strains were identified by searching through the VFDB database (Liu et al. 2019) via VFanalyzer (available at http://www.mgc.ac.cn/cgi‐bin/VFs/v5/main.cgi) and ARIBA run with the default parameters. Identified virulence genes were then curated by manual inspection of the pangenome protein clusters. Plasmidfinder (Carattoli et al. 2014) was used to evaluate the presence of plasmid replication origins.

### 2.9 Identification of *bla*_*CARB*_ Alleles, Comparative CARB Protein Sequence Analysis and Genetic Context of *bla*_*CARB*_

Previously unreported *bla*_*CARB*_ genes identified were submitted to NCBI for new β‐lactamase alleles designation. New alleles were designated as *bla*_*CARB‐59*_ (NCBI accession PQ246093, identified in VC41 strain), *bla*_*CARB‐60*_ (NCBI accessions PQ246094 in VC12, VC36, VC92 and VC95 and PQ246095 in VC77), *bla*_*CARB‐61*_ (NCBI accession PQ246096, in VC84) and *bla*_*CARB‐62*_ (NCBI accession PQ246097, in VC58). All the nucleotide sequences predicted by the genome annotation or the antimicrobial resistance gene analysis as encoding *bla*_*PSE*_ or *bla*_*CARB*_ β‐lactamases were obtained and then aligned with a collection of *bla*_*CARB*_ genes available at the NCBI database using Clustal Omega, through the EMBL‐EBI Job Dispatcher sequence analysis tools framework (Madeira et al. 2024). The phylogenetic tree generated was used as an input for building a cladogram using ChiPlot's application tvBOT (Xie et al. 2023) to depict the relationship at a nucleotide level between different *bla*_*CARB*_ enzymes. From these annotated β‐lactamases, predicted amino acid sequences were obtained and aligned by Clustal Omega MSA tool (Madeira et al. 2024), from which a percentage identity matrix was built. The β‐lactamases showing the highest homology were chosen for the alignment visualisation in SnapGene v7.2.1 software (www.snapgene.com) to identify conserved and polymorphic sites. The genetic context of *bla*_*CARB*_ was determined by analysing the open reading frames (ORFs) present in all contigs containing *bla*_*CARB*_ using NCBI Open Reading Frame Finder (www.ncbi.nlm.nih.gov/orffinder/) and BLASTN (https://blast.ncbi.nlm.nih.gov/Blast.cgi) tools.

### 2.10 Pangenome and Phylogenetic Analysis

The annotation of de novo assemblies was performed using Bakta (Schwengers et al. 2021). A total of 46 *V. cholerae* genomes were taken as an input for pangenome analysis through PanX (Ding et al. 2018), including *bla*_*CARB*_ positive strains detected in this study (*n* = 9) and the NCBI genome database (*n* = 6), as well as another 31 *V. cholerae* genomes retrieved from publicly available databases (NCBI genomes and PathogenWatch v22.3.8), as detailed in Table S6. Core genes were defined as those present in 99% of the strains, which yielded 1565 core genes. The core genome alignment containing only informative sites was then used to construct a mid‐point rooted Maximum‐Likelihood phylogeny by IQ‐TREE (Nguyen et al. 2015) (1000 bootstrap replicates), choosing the best substitution model (GTR+F+G4) according to Bayesian AIC criterion by the Model Finder parameter. The minimum phylogenetic tree branch support was 57%, with an average support of 95.06%. Bayesian analysis of population structure was conducted on the core genome alignment and phylogenetic tree using the FastBAPS algorithm (Tonkin‐Hill et al. 2019). Visualisation of the consensus phylogenetic tree was done by ChiPlot's application tvBOT (Xie et al. 2023).

### 2.11 Rabbit Ileal Loop Assays

Rabbit ileal loop assays based on the method by De Chaterjee (De and Chatterje 2010) were performed as previously described (Saka et al. 2008) and in accordance with institutional ethics requirements. Briefly, male New Zealand white rabbits (2.0–2.5 kg) were starved for 48 h prior to surgical procedures, for which animals were anaesthetised subcutaneously (ketamine 40 mg/kg and acepromazine 5 mg/kg). Midline incisions were done, small intestines were withdrawn and ligated approximately 10 cm from the ileocecal region, such that 10–12 loops of 6–8 cm separated by inter‐loop segments of 2–3 cm were obtained in each animal, preserving intestinal blood supply. Loops were inoculated with 1 mL of log‐phase cultures of each bacterial strain suspended in PBS 1× adjusted to 10^5^ CFU/mL. In each animal, one loop inoculated with PBS 1× alone as negative was included. At 18 h post‐inoculation, rabbits were euthanised and their intestines were removed. The volume of fluid accumulated within each loop was measured and expressed as the ratio of volume (ml)/length (cm) (FA ratio). Each strain was tested in at least three animals (three biological replicates per strain). As positive and negative controls, a CT‐producing *V. cholerae* O1 ‘El Tor’ (strain EP1/151) and a non‐virulent *E. coli* (strain DH5α) were processed in each animal.

## 3 Results

### 3.1 Origin, Virulence Genes and Antimicrobial Resistance Profile of Environmental *V. cholerae* Non‐O1/Non‐O139

**FIGURE 1:**
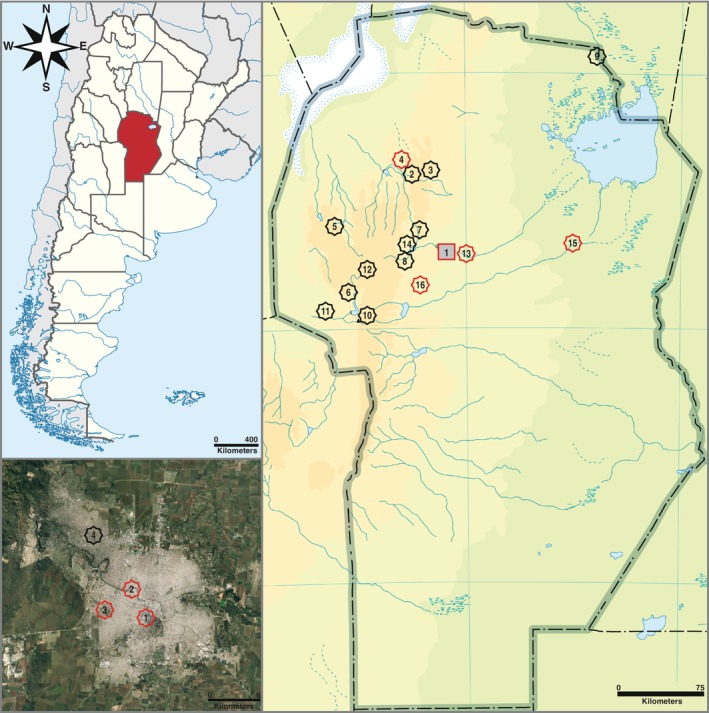
Geographical origin of environmental *V. cholerae* non‐O1/non‐O139 strains. Upper left panel: Map of Argentina with Córdoba province highlighted in red. Right panel: Map of Córdoba province showing the geographical location of all environmental *V. cholerae* non‐O1/non‐O139 isolates included in this study (*n* = 60). The grey square indicates the capital city of Córdoba (37 isolates), while all other locations are indicated by 8‐point stars (23 isolates). Red borders specify the presence of ampicillin‐resistant strains. Location names are signified by numbers, as follows: Córdoba city (1, 37 isolates); Dolores river (2, 2 isolates); Calabalumba river (3, 1 isolate); San Marcos river (4, 1 isolate); Salsacate river (5, 1 isolate); Los Sauces river (6, 1 isolate); Cosquín river (7, 1 isolate); San Antonio river (8, 1 isolate); Dulce river (9, 2 isolates); Los Molles river (10, 1 isolate); Los Sauces river (11, 2 isolates); Mina Clavero river (12, 1 isolate); Suquía river at Chacra La Merced (13, 4 isolates); San Roque dam (14, 3 isolates); Xanaes river (15, 1 isolate); Anisacate river (16, 1 isolate). Lower left panel: Map showing the origin of strains isolated in Córdoba city metropolitan area, as indicated by 8‐point stars. Red borders specify the presence of ampicillin‐resistant strains. Location names are signified by numbers, as follows: La Cañada stream (1, 15 isolates); Suquía river (2, 6 isolates); South master channel (3, 13 isolates); North master channel (4, 3 isolates).

A total of 60 *V. cholerae* non‐O1/non‐O139 isolates were obtained from 16 different freshwater sources (rivers, channels and a dam) located throughout the province of Córdoba, in the central region of Argentina (Figure 1): 37 strains were isolated from samples obtained in the city of Córdoba metropolitan area, 20 strains from 14 different rivers and 3 strains from a dam located approximately 40 km west of the city of Córdoba. As expected, PCR detection of virulence genes indicated that all *V. cholerae* non‐O1/non‐O139 strains (100%) were positive for the transcriptional activator ToxR/CadC (*toxR*) and *V. cholerae* cytolysin (*hlyA*), which are widely distributed in *V. cholerae* (Rivera et al. 2001), and none (0%) were positive for CT subunit A (*ctxA*), accessory cholera enterotoxin (*ace*), zonula occludens (*zot*) and toxin co‐regulated pilus subunit A (*tcpA*) (Table S5). Interestingly, among all antibiotics tested by disk diffusion (ampicillin, cefazolin, tetracycline, gentamicin, trimethoprim‐sulfamethoxazole, chloramphenicol and ciprofloxacin), the only phenotypic resistance detected was to ampicillin, present in 16% of the strains (Table S1). This prompted us to further investigate the mechanism mediating ampicillin resistance in these strains.

### 3.2 Genomic Analysis of Ampicillin‐Resistant *V. cholerae* Non‐O1/Non‐O139 Strains Identifies New CARB β‐Lactamase Variants

To identify the genetic basis of ampicillin resistance, we performed WGS analysis of all ampicillin‐resistant strains (*n* = 10). First, we carried out species‐level identification by rMLST and found that these strains were indeed *V. cholerae* except for VC84, which was identified as the newly recognised closely related species *V. paracholerae* (Islam et al. 2021). By MLST analysis, we found that all ampicillin‐resistant *V. cholerae* isolates corresponded to previously unreported ST profiles (assigned as new STs 1555, 1556 and 1558 to 1563 upon submission to the PubMLST database, Table 1). Unexpectedly, isolates VC92 and VC95 shared the same ST profile (ST1563), even though they were isolated on different dates and from different sampling locations. Consistent with phenotypic antibiotic resistance results, the resistome analysis revealed that all these strains harboured CARB β‐lactamases (Table 1) and lacked other acquired antibiotic resistance genes, as well as plasmid replication origins associated with antibiotic resistance (data not shown). The CARB family of β‐lactamases currently includes approximately 60 members, several of which have been identified in *Vibrio* species, including *V. cholerae* (Naas et al. 2017). CARB enzymes are class A β‐lactamases characterised by preferential hydrolysing activity against carbenicillin and ticarcillin (Bush and Jacoby 2010). DNA sequence analysis revealed that two strains (VC3 and VC97) carried *bla*_*CARB‐7*_, previously reported in an environmental *V. cholerae* non‐O1/non‐O139 isolate in Argentina (Melano et al. 2002), while the other strains carried *bla*_*CARB*_ alleles encoding previously unreported CARB variants, designated as *bla*_*CARB‐59*_ (strain VC41), *bla*_*CARB‐60*_ (VC12, VC36, VC77, VC92 and VC95), *bla*_*CARB‐61*_ (strain VC84) and *bla*_*CARB‐62*_ (strain VC58), as detailed in Table 1. Next, we investigated the susceptibility profile of the strains to a panel of different β‐lactam antibiotics. *V. cholerae* strains carrying *bla*_*CARB*_ determinants exhibited higher MIC values for ticarcillin and ampicillin, ranging from 32 to 512 μg/mL. These MICs were substantially reduced by 16‐ to 128‐fold in the presence of the β‐lactamase inhibitor clavulanic acid. Additionally, these strains showed low MICs against piperacillin and cefazolin, both ranging from 1 to 4 μg/mL, consistent with the expected susceptibility pattern for CARB β‐lactamases (Table 1). In contrast, *V. cholerae* non‐O1/non‐O139 strains lacking *bla*_*CARB*_ were uniformly susceptible to all β‐lactams tested. Furthermore, ampicillin‐hydrolysing bands with isoelectric point values ranging 5.2–5.4 were observed in all *bla*_*CARB*_ strains by analytical isoelectric focusing (Table 1). These results indicate that *bla*_*CARB*_ genes confer the ampicillin resistance phenotype observed.

**TABLE 1:** Antimicrobial susceptibility profile of *V. cholerae* strains to selected β‐lactam antibiotics.

Strain ID	ST^a^	*bla*_*CARB*_ alleles	Isoelectric point^b^		MIC (μg/mL)								
			Predicted	Observed	AMP	AMS	AMC	TIC	TIC/CLAV	PIP	CEF	FOX	CTX
VC3	1555	*bla*_*CARB‐7*_	5.5	5.4	256	2	2	512	4	0.5	4	2	< 0.12
VC97	1558	*bla*_*CARB‐7*_	5.5	5.4	32	2	2	64	2	2	1	1	< 0.12
VC41	1561	*bla*_*CARB‐59*_	5.5	5.4	64	1	1	32	2	4	0.5	1	< 0.12
VC12	1556	*bla*_*CARB‐60*_	5.5	5.4	64	4	4	64	4	4	1	2	< 0.12
VC36	1560	*bla*_*CARB‐60*_	5.5	5.4	64	4	4	128	8	4	0.5	1	< 0.12
VC77	1559	*bla*_*CARB‐60*_	5.5	5.4	64	1	2	64	4	0.5	1	1	< 0.12
VC92	1563	*bla*_*CARB‐60*_	5.5	5.4	64	2	4	64	4	2	0.5	1	< 0.12
VC95	1563	*bla*_*CARB‐60*_	5.5	5.4	128	4	4	128	4	4	1	1	< 0.12
VC84^c^	NA	*bla*_*CARB‐61*_	5.5	5.4	512	8	32	512	32	1	4	2	< 0.12
VC58	1562	*bla*_*CARB‐62*_	5.3	5.2	64	2	2	32	1	1	1	1	< 0.12
VC4	ND	Negative	NA	NA	1	1	1	1	1	< 0.12	0.25	0.5	< 0.12
VC44	ND	Negative	NA	NA	1	1	1	0.5	0.5	< 0.12	0.5	1	< 0.12
VC20	ND	Negative	NA	NA	1	1	2	1	1	< 0.12	0.5	1	< 0.12
VC29	ND	Negative	NA	NA	0.25	1	1	0.5	0.5	< 0.12	0,25	1	< 0.12
VC52	ND	Negative	NA	NA	2	2	4	1	1	0.25	2	1	< 0.12

Abbreviations: AMC, amoxicillin‐clavulanic acid; AMP, ampicillin; AMS, ampicillin‐sulbactam; CEF, cefazolin; CTX, cefotaxime; FOX, cefoxitin; MIC, minimal inhibitory concentrations; ND, not determined; PIP, piperacillin; TIC, ticarcillin; TIC/CLAV, ticarcillin‐clavulanic acid.

^a^ ST, sequence type by MLST.

^b^ Predicted isoelectric points were determined using the bioinformatic tool http://isoelectric.org/. Observed isoelectric points were determined by analytical isoelectric focusing as specified in the Experimental Procedures section.

^c^ Identified as *Vibrio paracholerae* by rMLST analysis.

**FIGURE 2:**
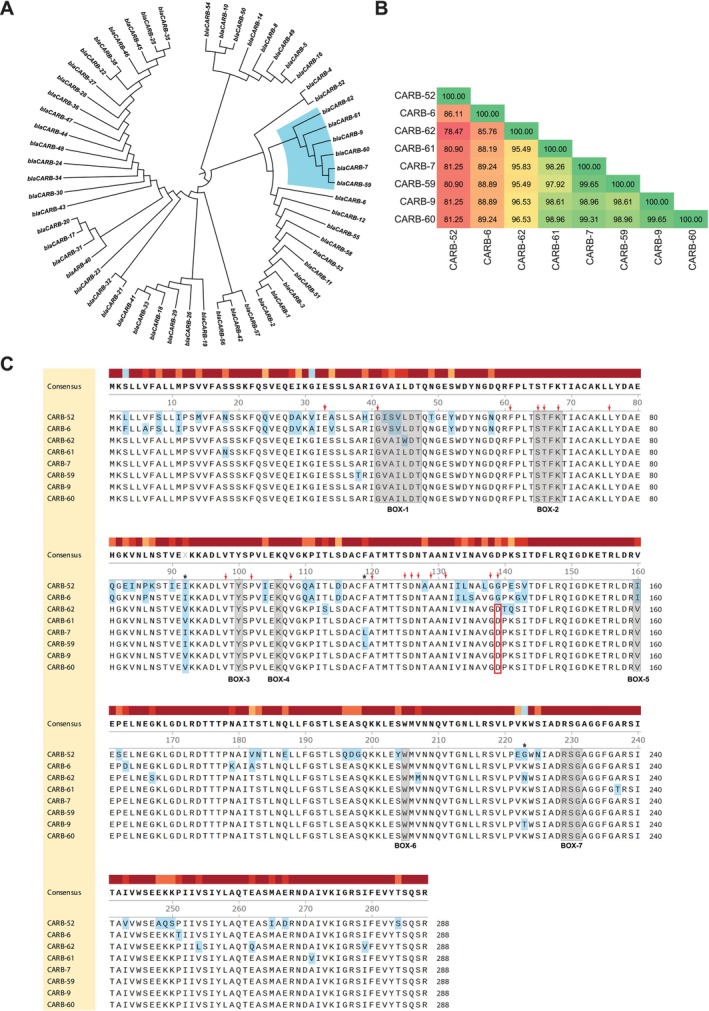
Cladogram of *bla*_*CARB*_ genes and protein sequence analysis of CARB β‐lactamases. (A) A cladogram was built from a multiple sequence alignment of *bla*_*CARB*_ genes using Clustal Omega (ClustalO). The branch containing *bla*_*CARB*_ alleles identified in this study is highlighted in light blue. (B) A percentage identity matrix (PIM) was generated using ClustalO from a multiple sequence alignment of CARB β‐lactamases included in the highlighted branch plus CARB‐6 and CARB‐52 (the closest CARB enzymes from the nearest branches). (C) Multiple sequence alignment of selected CARB β‐lactamases protein sequences using ClustalO. The shadowed boxes (1–7) represent amino acid motifs conserved in penicillin‐recognising enzymes. Red arrows indicate conserved residues specific for class A β‐lactamases. Polymorphic residues are highlighted in light blue. The red box highlights the substitution of glycine (G) to aspartic acid (D) at position 139 (equivalent to position 144 based on Ambler classification). Polymorphic sites at amino acid positions 92, 119 and 223 (103, 130 and 234 in the Ambler scheme) are indicated with asterisks.

BLAST analysis at the nucleotide level showed that the previously unreported *bla*_*CARB*_ variants were encoded in 867 bp ORFs sharing 97.0%–99.9% and 96.9%–99.2% identities with *bla*_*CARB‐7*_ and *bla*_*CARB‐9*_, respectively. To analyse the relatedness of the *bla*_*CARB*_ genes detected with all previously reported *bla*_*CARB*_ alleles, a cladogram was generated from a multiple alignment of *bla*_*CARB*_ coding sequences. As observed in Figure 2A, the new *bla*_*CARB*_ alleles were grouped in the same branch together with *bla*_*CARB‐7*_ and *bla*_*CARB‐9*_, also described in environmental *V. cholerae* non‐O1/non‐O139 strains from Argentina (Melano et al. 2002; Petroni et al. 2004). The cladogram showed that *bla*_*CARB‐59*_ and *bla*_*CARB‐60*_ formed a sub‐branch together with *bla*_*CARB‐7*_ and shared a common ancestor with *bla*_*CARB‐9*_, while the other two alleles (*bla*_*CARB‐61*_ and *bla*_*CARB‐62*_) were slightly less related. The next closest *bla*_*CARB*_ alleles corresponded to *bla*_*CARB‐6*_ (Choury et al. 1999) and *bla*_*CARB‐52*_ (NCBI accession: MN339507.1), both detected in France in a *V. cholerae* non‐O1/non‐O139 and a *Pseudomonas aeruginosa* clinical isolate, respectively. Noticeably, all *bla*_*CARB*_ alleles described in *V. cholerae* non‐O1/non‐O139 strains from Argentina were grouped in a separate sub‐clade sharing a common ancestor with all CARB‐1‐like enzymes. Comparative analysis of the amino acid sequences showed that the new CARB variants shared 95.83% to 99.65% protein identities with CARB‐7 and CARB‐9, with CARB‐59 showing higher identity with CARB‐7 compared to CARB‐9 (99.65% vs. 98.61%) while the opposite was observed for CARB‐60 (99.31% vs. 99.65%), as shown in Figure 2B. In line with the cladogram of *bla*_*CARB*_ genes, the new variants CARB‐61 and CARB‐62 presented slightly lower protein identities with CARB‐7 (95.83% to 98.26%) and CARB‐9 (96.53% to 98.61%) in comparison with CARB‐59 and CARB‐60. Also in agreement with the cladogram, among the newly identified CARB enzymes, CARB‐62 was the one presenting the lowest protein identities with CARB‐7 and CARB‐9 (95.83% and 96.53%, respectively). The next closest CARB enzymes CARB‐6 (Choury et al. 1999) and CARB‐52 showed 85.76%–89.24% and 78.47%–81.25% protein identities with the newly identified CARB variants, respectively (Figure 2B). To further characterise the new CARB variants, we carried out multiple sequence alignments of the 288 amino acids‐long CARB‐59 to CARB‐62 enzymes and included CARB‐6 and CARB‐52 in the analysis. As shown in Figure 2C, the new variants contained all seven conserved amino acid boxes, as well as the active site tetrad ‘STFK’ and specific conserved amino acid residues characteristically found in serine‐dependent, penicillin‐recognising class A β‐lactamases (Joris et al. 1988). Interestingly, all new CARB enzymes shared with CARB‐7 (Melano et al. 2002) and CARB‐9 (Petroni et al. 2004), the presence of D instead of G at amino acid 139 (corresponding to position 144 in the Ambler classification scheme (Ambler et al. 1991) for class A β‐lactamases) in the last of the 18 conserved amino acid residues. Another interesting observation was related to the polymorphisms in amino acid positions 92, 119 and 223 (103, 130 and 234 in the Ambler scheme), which differentiate CARB‐7 from CARB‐9 (Petroni et al. 2004). CARB‐59 shared with CARB‐7 ‘I‐L‐K’ in those positions, as opposed to ‘V‐F‐K’ in CARB‐60 and CARB‐61, ‘V‐F‐N’ in CARB‐62 and ‘V‐F‐T’ in CARB‐9 (Figure 2C). Thus, CARB‐60/−61 and CARB‐62 carry a unique combination of polymorphisms in those positions, separating them from CARB‐7/−59 and CARB‐9.

### 3.3 *bla*_*CARB*_ Alleles Are Flanked by *V. cholerae* Repeats (VCR) Sequences, Suggesting Their Association With the *V. cholerae* Superintegron

**FIGURE 3:**
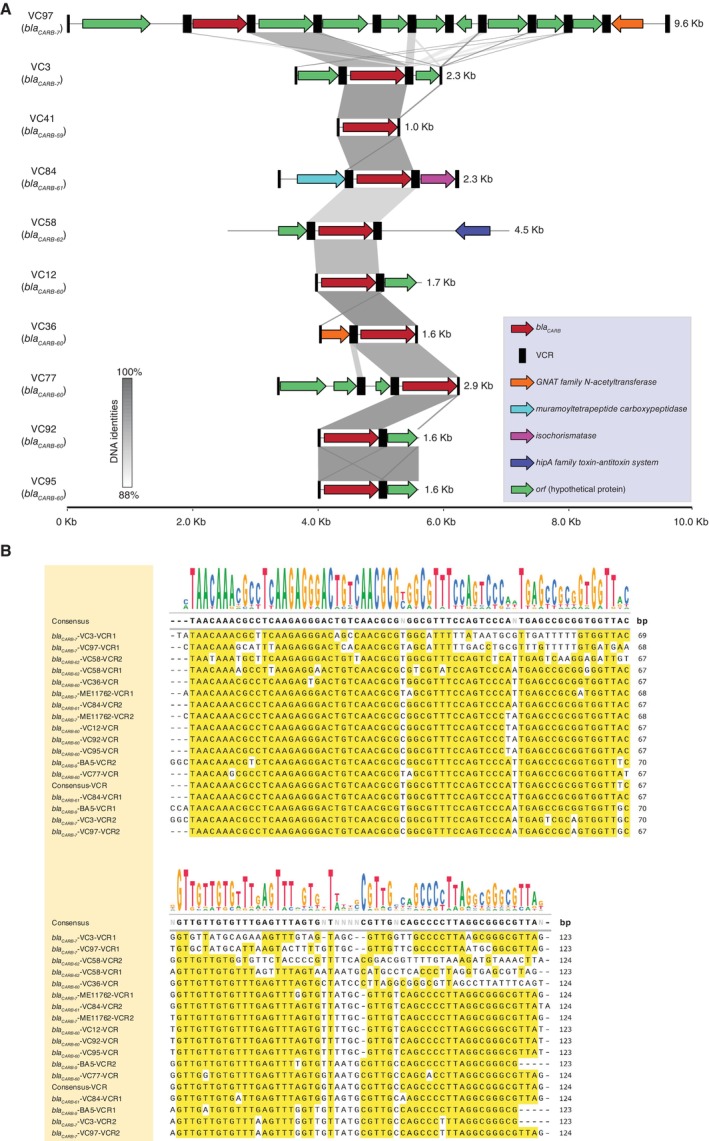
Genetic context of newly identified *bla*_*CARB*_ variants. (A) The contigs containing *bla*_*CARB*_ genes identified by AMRFinderPlus in *V. cholerae* strains were extracted, along with their respective annotations, for comparative genomic visualisation using pyGenomeViz software. The comparison of the nucleotide sequences was carried out using BLASTN. Predicted ORFs and *Vibrio cholerae* repeats (VCRs) are indicated. DNA identities ≥ 88% are highlighted. DNA sizes are expressed in kilobases (kb). (B) Multiple sequence alignment of complete VCR DNA sequences flanking *bla*_*CARB*_ in *V. cholerae* strains, including the ones corresponding to the first description of *bla*_*CARB‐7*_ (Melano et al. 2002), *bla*_*CARB‐9*_ (Petroni et al. 2004) and the consensus VCR sequence (Barker et al. 1994). Base identities are highlighted in yellow. DNA size is indicated in base pairs (bp).

*V. cholerae* encodes a large integron‐like structure, termed the *V. cholerae* superintegron, which spans approximately 130 kb, constituting about 3% of the genome and 10% of chromosome II (Mazel et al. 1998; Heidelberg et al. 2000; Mazel 2006). A defining feature of this superintegron is the presence of numerous repetitive 123–126 bp DNA sequences, known as VCRs, which exhibit imperfect symmetry and flank the ORFs within the superintegron (Mazel et al. 1998; Mazel 2006; Barker et al. 1994). Previous studies have shown that both *bla*_*CARB‐7*_ and *bla*_*CARB‐9*_ are flanked by VCRs, suggesting their location within the *V. cholerae* superintegron (Melano et al. 2002; Petroni et al. 2004). Thus, we searched for VCR consensus sequences within the contigs containing *bla*_*CARB*_ genes. Our analysis found evidence of VCRs flanking all *bla*_*CARB*_ genes. As illustrated in Figure 3, BLAST analysis and multiple sequence alignment of VCR sequences confirmed that the genetic context of *bla*_*CARB*_ genes is consistent with the *V. cholerae* superintegron, with VCRs flanking *bla*_*CARB*_ and other predicted ORFs primarily encoding hypothetical proteins. This is particularly evident in VC97, where 11 predicted ORFs, including *bla*_*CARB‐7*_, are flanked by VCRs (Figure 3A). The smaller contig corresponded to VC41, which only contained the complete ORF corresponding to *bla*_*CARB‐59*_ flanked by partially covered VCRs. In the case of VC58, although *bla*_*CARB‐62*_ was flanked by VCRs, no additional VCRs were identified in the contig. Thus, the association of *bla*_*CARB‐59*_ and *bla*_*CARB‐62*_ with the superintegron, while suggestive, remains less clear.

### 3.4 Virulence Features of Environmental *V. cholerae* Strains Harbouring *bla*_*CARB*_

**FIGURE 4:**
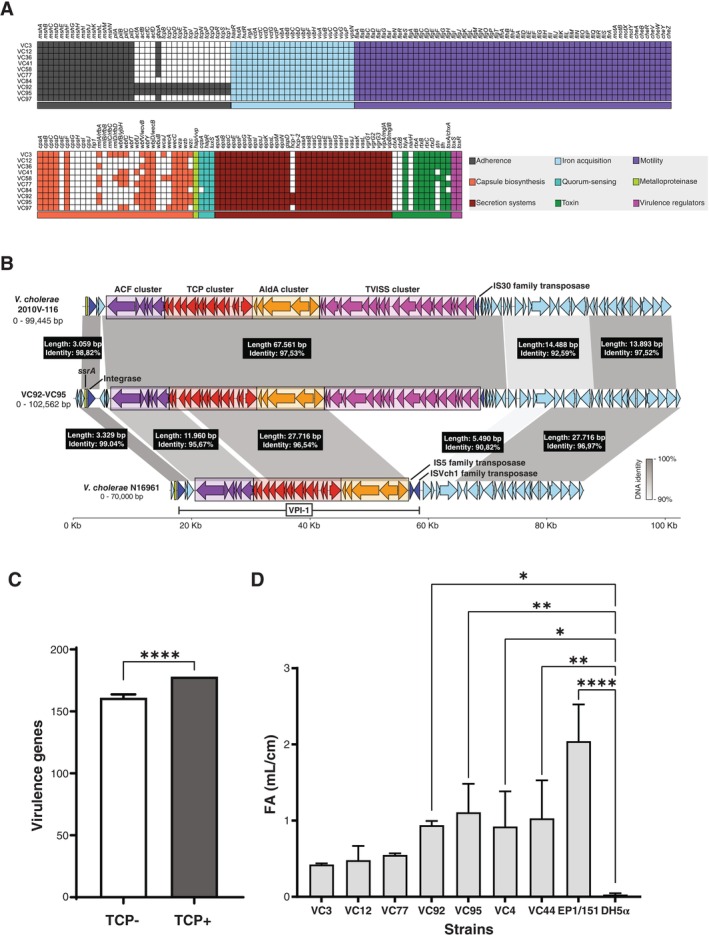
Virulome and enterotoxic potential of environmental *V. cholerae* strains encoding *bla*_*CARB*_. (A) Virulome profile of environmental *V. cholerae* strains encoding *bla*_*CARB*_. *V. cholerae* non‐O1/non‐O139: VC3 (*bla*_*CARB‐7*_), VC12 (*bla*_*CARB‐60*_), VC36 (*bla*_*CARB‐60*_), VC41 (*bla*_*CARB‐59*_), VC58 (*bla*_*CARB‐62*_), VC77 (*bla*_*CARB‐60*_), VC92 (*bla*_*CARB‐60*_), VC95 (*bla*_*CARB‐60*_), VC97 (*bla*_*CARB‐7*_); *V. paracholerae*: VC84 (*bla*_*CARB‐61*_). Virulence genes were grouped by functional categories, as indicated. (B) Alignment of coding sequences identified in the context of the VPI‐1 genomic region in strains VC92 and VC95, with *V. cholerae* O1 ‘El Tor’ N16961 (accession: GCF_900205735.1; chromosome 1) and *V. cholerae* non‐O1/non‐O139 2010V‐116 strain (accession: GCF_012275105.1; chromosome 1) performed by pyGenomeViz software. DNA identities ≥ 90% are highlighted. Coloured arrows within squares indicate genes corresponding to ACF, TCP, AldA or TVISS clusters. TVISS clusters found in the context of VPI‐1 were identified as subclass i5 using SecReT6 v3 online tool. Integrases, insertion sequences (IS) and transfer‐messenger RNA sequences (*ssrA*) are shown. DNA sizes are indicated in base pairs (bp) and kilobase pairs (Kbp). (C) Comparison of the total number of virulence genes identified in *V. cholerae* strains encoding *bla*_*CARB*_ grouped by TCP‐ (VC3, VC12, VC36, VC41, VC58, VC77, VC84 and VC97) and TCP+ (VC92 and VC95). Bars represent the mean and standard deviation of the number of virulence genes identified in each group (TCP+: 167,0 ± 0, TCP‐ 147,8 ± 3,2). Statistical significance was determined by unpaired *t*‐test (****p < 0.0001). (D) Rabbit ileal loop assays were performed to evaluate enterotoxigenicity of a randomly selected subset of *V. cholerae* strains encoding *bla*_*CARB*_ (VC3, VC12, VC77, VC92 and VC95). For comparison, two CT‐negative *V. cholerae* non‐O1/non‐O139 strains (VC4 and VC44) isolated from patients suffering cholera‐like diarrheal disease were included. A CT‐producing *V. cholerae* O1 ‘El Tor’ strain (EP1/151) and a non‐virulent *E. coli* strain (DH5α) were used as positive and negative controls, respectively. Bars represent the mean and standard deviation of the fluid accumulation ratio (FA) obtained for each strain out of 3 independent experiments. Statistically significant differences compared to DH5α strain as determined by ordinary ANOVA and Dunnett's multiple comparisons test are indicated (*p < 0.05; **p < 0.01; ****p < 0.0001).

*V. cholerae* non‐O1/non‐O139 strains are typical inhabitants of aquatic environments and generally do not produce the major virulence factors of cholera pathogenesis, CT and the toxin co‐regulated pilus (TCP). However, they can infect humans, causing diarrheal diseases, including life‐threatening cholera‐like syndrome. As previously mentioned, initial characterisation using PCR detection of selected virulence genes indicated that all *V. cholerae* strains harbouring *bla*_*CARB*_ lacked CT and TCP, as expected (Table S5). To achieve a more comprehensive characterisation of the virulent potential of these strains, we elucidated their virulome. As shown in Figure 4A, all strains uniformly carried *msh* and *pil* genes, which encode MSHA and ChiRP pili, respectively. Both are type IV fimbrial adhesins important for attachment and biofilm formation (Teschler et al. 2015; Fullner and Mekalanos 1999; Paranjpye and Strom 2005). Similarly, they all encoded iron acquisition systems, including vibriobactin (*vib* genes), enterobactin receptors (*irgA*, *vctA*), the periplasmic ABC transporter gene clusters *vctPDGC* and *viuPDGC* for the transport of both vibriobactin and enterobactin, and heme receptors (*hasR*, *hutA*, *hutR*) (Wyckoff et al. 2007). Also, the complete repertoire of genes required for flagellum biosynthesis (Zhu et al. 2013; Minamino and Imada 2015) was found in the motility category for all isolates. The presence of virulence genes involved in capsule biosynthesis was also generalised, as well as genes related to quorum sensing (*cqsA*, *luxS*, *hapR*), type II (*eps* and *gspD*) and type VI (*vas*, *vgrG*, *hcp* and *vip*) secretion systems. Regarding the presence of toxin‐coding genes and virulence regulators, none of the strains carried CT (*ctxAB*) but all of them were positive for *V. cholerae* cytolysin (*hlyA*) and the *toxRS* regulators, in agreement with PCR results. In addition, all strains harboured the gene cluster encoding the RTX toxin (*rtxABCD*) (Lin et al. 1999) and the thermolabile hemolysin (*tlh*), which is widely distributed in *V. cholerae* (Wan et al. 2019). Interestingly, most isolates also encoded the Cholix exotoxin (*toxA/chxA*) (Lugo and Merrill 2015), while the heat‐stable enterotoxin (*stn*) (Ogawa et al. 1990) was present in 4 of the strains (4 out 10). Remarkably, strains VC92 and VC95, which shared the same sequence type by MLST (ST1563, Table 1), were both positive for the TCP gene cluster (Figure 4A,B), in apparent disagreement with negative results for the *tcpA* gene by PCR (Table S5). However, DNA sequence analysis revealed polymorphisms in the target sequence of one of the primers used for PCR detection of *tcpA* in these strains (data not shown). To characterise the genomic region encoding the TCP gene cluster in VC92 and VC95, we carried out a DNA alignment with toxigenic *V. cholerae* O1 ‘El Tor’ N16961 and non‐toxigenic *V. cholerae* non‐O1/non‐O139 2010V‐116 (Figure 4B). Interestingly, our analysis revealed that both VC92 and VC95 harbour the *V. cholerae* pathogenicity island VPI‐1, a horizontally acquired DNA fragment approximately 41.3 kb in size, found in all pandemic strains (Kumar et al. 2020). This genetic element facilitates the expression of the TCP pilus, which plays a dual role: it mediates adherence to intestinal epithelial cells and enables the acquisition of the CTXϕ bacteriophage, which carries the CT genes (Manning 1997). In addition, a type VI secretion system (TVISS) large cluster was found in the context of VPI‐1 in VC92 and VC95 strains. This macromolecular harpoon‐like system, universally present in *V. cholerae* , spans the inner membrane, periplasmic space and outer membrane of *V. cholerae* cells, allowing for secretion of effector proteins important for both pathogenesis and the ecology of this bacterium (Crisan and Hammer 2020; Unterweger et al. 2014; Pukatzki et al. 2006). DNA BLAST analysis indicated that this arrangement is uncommon, since the only *V. cholerae* genomes with the same genetic structure found in the NCBI database were a non‐toxigenic strain 2010V‐116 isolated in Haiti (Figure 4B) and strain ICDC‐VC702 from China (accession: NZ_CP080462, data not shown). Remarkably, this TVISS cluster associated with VPI‐1 corresponded to TVISS subclass i5, as predicted by the SecReT6 bioinformatic tool (Zhang et al. 2023). However, these strains also carried a TVISS cluster subclass i1 in another genomic location, perfectly aligned with a region of chromosome II encoding the same cluster in *V. cholerae* strains N16961 and 2010V‐116 (Figure S1). Thus, VC92 and VC95 carried two different subclasses of the TVISS large cluster, one associated with the VPI‐1 element, and likely located in a genomic region of chromosome II. To further evaluate the virulence potential of *V. cholerae* strains, we quantified the presence of virulence genes. This analysis showed that TCP+ strains VC92 and VC95 possessed a significantly higher average number of virulence genes compared to the rest of the strains (Figure 4C). Additionally, in vivo experiments demonstrated that VC92 and VC95 induced greater fluid accumulation in rabbit ileal loops, similar to the response elicited by *V. cholerae* non‐O1/non‐O139 clinical strains isolated from patients with cholera‐like diarrheal disease (Figure 4D). These findings suggest that environmental *V. cholerae* strains harbouring *bla*_*CARB*_ contain a diverse array of virulence genes and underscore that strains with the TCP gene cluster exhibit enhanced enterotoxigenicity.

### 3.5 Phylogenetic Analysis of *V. cholerae* Strains

**FIGURE 5:**
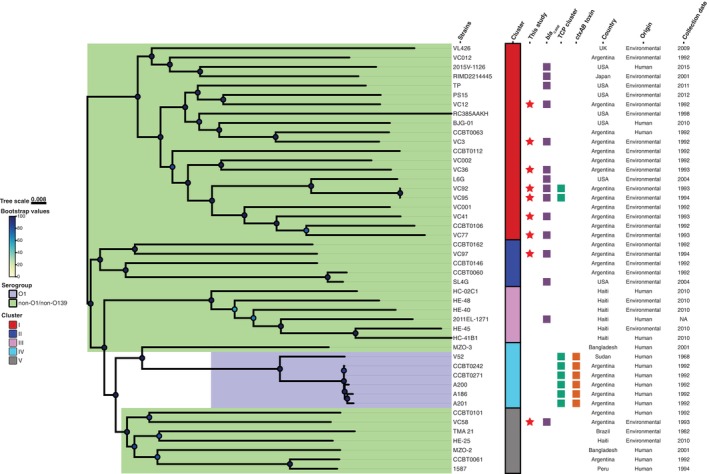
*Vibrio cholerae* core‐genome Maximum‐Likelihood phylogenetic tree. *V. cholerae* isolates encoding *bla*_*CARB*_ from this study (*n* = 9) and the NCBI genome database (*n* = 6), plus those of additional *V. cholerae* strains (*n* = 31) were employed to build a core‐genome midpoint rooted SNP phylogenetic tree using IQ‐TREE (46 strains total). All branches had a support value over 67% (average branch support: 95.06%). Bootstrap values, serotypes, FastBAPS predicted clusters, date and location information, and the presence/absence of genes of interest are indicated. Additional information about the strains included is available in Table S6.

To investigate the genetic relationships of *V. cholerae* strains encoding *bla*_*CARB*_ in a global context, a phylogenetic analysis of core‐genome SNPs on 46 *V. cholera* isolates from different sources, times and locations was constructed (Figure 5). The selected genomes were retrieved from public databases and included strains from environmental (*n* = 29) and human (*n* = 17) sources, isolated from Argentina (*n* = 25), Haiti (*n* = 7), United States of America (USA) (*n* = 7), Bangladesh (*n* = 2), Brazil, Japan, Peru, Sudan and United Kingdom (UK) (*n* = 1 each). The analysis revealed a high level of genetic diversity, with each strain presenting over 30,000 SNPs in core genes when compared to one another (data not shown). Using the FastBAPS algorithm on the mid‐point rooted phylogenetic tree, five distinct clusters were identified. Four of these clusters (I, II, III, V) contained non‐O1/non‐O139 serogroups, while all O1 serogroups were grouped into a separate cluster (IV). Regarding the *bla*_*CARB*_‐positive strains identified in this study, seven out of nine strains (VC3, VC12, VC36, VC41, VC77, VC92, VC95) shared a more recent common ancestor and were grouped in cluster I, along with four additional *bla*_*CARB*_‐positive strains isolated in the USA (*n* = 3) and Japan (*n* = 1). Three of these were of environmental origin, while one from the USA was of human origin. The fact that 11 out of 14 *bla*_*CARB*_‐positive strains grouped in cluster I may suggest a potential association of this global cluster with an increased ability to acquire *bla*_*CARB*_. The remaining two strains were found in separate clusters: VC97 in cluster II, which also included a *bla*_*CARB*_‐positive strain from the USA, and VC58 in cluster V. Clusters V and IV shared a more recent common ancestor, suggesting a closer genetic relationship of strain VC58 with O1 serogroups. Additionally, a *bla*_*CARB*_‐positive strain from Haiti was found in cluster III. In summary, while most local *bla*_*CARB*_‐encoding strains were grouped in cluster I, no clear associations were observed, reflecting the high genetic diversity of the isolates.

## 4 Discussion

This study offers a comprehensive characterisation of *V. cholerae* non‐O1/non‐O139 strains from various natural freshwater bodies in central Argentina. Ampicillin resistance was found to be the most prevalent antibiotic resistance phenotype, despite these strains being generally susceptible to antibiotics. A limitation of our study is that it is focused on strains isolated from 1991 to 1994, which may not reflect the current situation, particularly regarding antibiotic resistance. However, our finding is consistent with another analysis of environmental *V. cholerae* non‐O1/non‐O139 strains from other geographic areas in Argentina obtained from 2003 to 2005, which also identified ampicillin resistance as the more relevant resistance phenotype (Fraga et al. 2007). This is intriguing since the environmental sources of these strains and our strains are unlikely to experience significant anthropogenic β‐lactam pressure. Our WGS analysis identified that all ampicillin‐resistant strains carried *bla*_*CARB*_ genes as the only determinant of acquired resistance to β‐lactams. Accordingly, the MIC profile to a panel of β‐lactam antibiotics was fully consistent with the presence of CARB β‐lactamases, due to higher activities against ticarcillin and ampicillin, reversed by clavulanic acid. Further DNA sequence and structural analysis identified four new *bla*_*CARB*_ alleles, designated as *bla*_*CARB59*_ to *bla*_*CARB‐62*_, which are related to *bla*_*CARB‐7*_ and *bla*_*CARB‐9*_ previously described in *V. cholerae* non‐O1/non‐O139 from Argentina (Melano et al. 2002; Petroni et al. 2004). Our findings agree with other investigators who have also linked ampicillin resistance in *V. cholerae* to the presence of *bla*_*CARB*_. For instance, a study on clinical *Vibrio* spp. isolates in Germany reported that 3 out of 28 (~11%) of *V. cholerae* non‐O1/non‐O139 strains isolated in 2018–2019 were ampicillin‐resistant, with 2 carrying *bla*_*CARB‐7*_ (Brehm et al. 2021). Another study focused on environmental *Vibrio* spp. from freshwater sources in Nigeria identified *bla*_*CARB*_ genes in 11% of the isolates, though it did not specify the *Vibrio* species or alleles involved or the isolation dates (Adesiyan et al. 2022). Additionally, ampicillin resistance was found to be among the most frequent resistances in *V. cholerae* non‐O1/non‐O139 from both clinical and environmental origins in Austria obtained from 2000 to 2015, reaching 24% and also associated with the presence of *bla*_*CARB*_ variants (Lepuschitz et al. 2019). Similar results were reported in Australia, where 23% of *V. cholerae* non‐O1/non‐O139 isolated from 1983 to 2020 harboured *bla*_*CARB‐9*_ (Bhandari et al. 2023). Together, these findings suggest that ampicillin resistance, mediated by CARB β‐lactamases, is a widespread feature in *V. cholerae* non‐O1/non‐O139 populations. This evidence supports that this resistance appears to be predominantly associated with various CARB β‐lactamases. Including our study, at least six different CARBs have been identified in *V. cholerae* : CARB‐6 (Choury et al. 1999), CARB‐7 (Melano et al. 2002), CARB‐9 (Petroni et al. 2004), and the newly described CARB‐59, CARB‐60, and CARB‐62. Plus, in this work we identified CARB‐61 in an isolate initially typed as *V. cholerae* and later re‐typed as *V. paracholerae* by rMLST analysis. Notably, except for CARB‐6, which was first identified in France, all other CARB variants linked to *V. cholerae* non‐O1/non‐O139 were reported in Argentina.

Regarding the genetic context of the *bla*_*CARB*_ genes encoding CARB‐59 to CARB‐62, we identified evidence of VCR sequences flanking all of them, strongly suggesting an association with the *V. cholerae* superintegron, as previously reported for *bla*_*CARB‐7*_ and *bla*_*CARB‐9*_ (Melano et al. 2002; Petroni et al. 2004). However, due to the small size (~1 kb) of the contig containing *bla*_*CARB‐59*_, the flanking VCR sequences were only partially covered. For *bla*_*CARB‐62*_, although the flanking VCR sequences were fully covered, no additional ORFs flanked by VCRs were identified in the contig. Therefore, while the presence of flanking VCR sequences for both *bla*_*CARB‐59*_ and *bla*_*CARB‐62*_ suggests their location in the *V. cholerae* superintegron, this evidence is not conclusive. In contrast, the contigs containing *bla*_*CARB‐60*_ and *bla*_*CARB‐61*_ also included other ORFs flanked by VCRs, indicating a more likely location within the superintegron, like the case of strains harbouring *bla*_*CARB‐7*_. Collectively, the genetic environment observed for these *bla*_*CARB*_ variants supports the hypothesis that they are cassette‐encoded β‐lactamases likely captured by the *V. cholerae* superintegron, as earlier proposed for *bla*_*CARB‐7*_ and *bla*_*CARB‐9*_ (Petroni et al. 2004).

Given the potential of *V. cholerae* non‐O1/non‐O139 strains to cause a range of human illnesses, including sporadic cases and outbreaks of diarrheal disease, ear infections, wound and soft tissue infections, bacteremia and sepsis (Arteaga et al. 2020; Baker‐Austin et al. 2017), we aimed to characterise their virulence features. In this sense, it is relevant to mention that the pathogenic mechanisms of *V. cholerae* non‐O1/non‐O139 remain poorly understood. An initial PCR‐based detection of key virulence genes revealed that, as expected, none of the strains carried CT. However, all harboured the *toxR* virulence regulator and *hlyA*, which encodes *V. cholerae* cytolysin. This virulence profile is typical for *V. cholerae* non‐O1/non‐O139 and aligns with numerous studies (Arteaga et al. 2020; Fraga et al. 2007; Bhandari et al. 2023; Schmidt et al. 2023; Schwartz et al. 2019; Ceccarelli et al. 2015; Luo et al. 2021; Zago et al. 2017; Gonzalez Fraga et al. 2009). Leveraging WGS, we detailed the virulome profile of the *bla*_*CARB*_‐positive strains, finding that they uniformly carried genes for vibriobactin, enterobactin receptors and heme receptors involved in iron acquisition. Notably, *V. cholerae* 's iron‐scavenging ability has been shown to facilitate its pathogenesis and environmental survival, highlighting a possible role for iron acquisition mechanisms in *Vibrio* virulence (Henderson and Payne 1994; Byun et al. 2020). Further, as detected in other studies, all strains carried genes for type IV fimbrial adhesins MSHA and ChiRP, capsule biosynthesis, quorum‐sensing, type II and type VI secretion systems (Arteaga et al. 2020; Bhandari et al. 2023; Schmidt et al. 2023; Ceccarelli et al. 2015), which may all directly and/or indirectly contribute to the virulent potential of these strains. Of note, type III secretion system genes, which have also been linked to increased virulence in *V. cholerae* non‐O1/non‐O139 strains (Arteaga et al. 2020; Zeb et al. 2019), were absent in all *bla*_*CARB*_‐positive strains from our study. Several toxin‐coding genes were identified in the strains, some of which are widely distributed. In addition to *V. cholerae* cytolysin, all strains harboured genes encoding RTX and TLH toxins. *V. cholerae* cytolysin is a pore‐forming exotoxin that induces various detrimental effects, including host cell lysis, apoptosis, vacuolization and autophagy in cell culture, as well as necrosis, apoptosis, fluid accumulation and shortening of intestinal villi in rabbit ileal loops (Saka et al. 2008; Ichinose et al. 1987; Zitzer et al. 1995; Figueroa‐Arredondo et al. 2001; Moschioni et al. 2002; Gutierrez et al. 2007). The RTX (repeats in toxin) toxin belongs to a group of large proteins known as multifunctional‐autoprocessing RTX toxins (MARTX) (Lin et al. 1999; Chatterjee et al. 2008; Coote 1992; Woida and Satchell 2018). In *V. cholerae* , the RTX toxin (encoded by the *rtxA* gene) is found in nearly all strains. It disassembles the host cell cytoskeleton through actin depolymerization, likely preventing early bacterial clearance in the intestine and promoting a more persistent infection (Cordero et al. 2006; Olivier et al. 2009; Satchell 2015). TLH, a hemolysin with lysophospholipase enzymatic activity, causes cytotoxic effects in various cell types in vitro (Wang et al. 2015). It is widely distributed not only in *V. cholerae* but also in other *Vibrio* species such as *V. parahaemolyticus* , *Vibrio anguillarum* , *V. vulnificus* , *V. alginolyticus* and *V. harveyi* (Vazquez‐Morado et al. 2021; Klein et al. 2014; Wang et al. 2007; Fu et al. 2020). Cholix toxin, encoded by *chxA/toxA*, is an ADP‐ribosyltransferase that targets the host cell's elongation factor 2, thereby blocking protein synthesis (Jorgensen et al. 2008). In our study, this gene was found in 8 out of 10 strains. Other studies have also reported the relatively common presence of *chxA* in *V. cholerae* non‐O1/non‐O139 strains with varied prevalences. For example, a study in Japan detected this gene in 27% of non‐O1/non‐O139 strains, while it was absent in O1 strains (Awasthi et al. 2013). Another study in Australia found *chxA* in 55% of *V. cholerae* non‐O1/non‐O139 strains (Bhandari et al. 2023). The *stn* gene encodes the heat‐stable enterotoxin NAG‐ST, a relatively small polypeptide with the ability to trigger calcium release from host cells' intracellular stores and activation of cGMP production (Ogawa et al. 1990; Hoque et al. 2001, 2003; Visweswariah et al. 1992). NAG‐ST has been associated with increased ability to cause diarrhoea (Morris Jr. et al. 1990). Our analysis identified the presence of *stn* in 4 out of 10 strains, suggesting that the production of NAG‐ST is not uncommon in local environmental *V. cholerae* non‐O1/non‐O139 strains. However, prevalences of *stn* seem to vary widely. For instance, surveys reported 21% in the USA (Ceccarelli et al. 2015), 9.6% in Thailand (Dalsgaard et al. 1995), 3.4% in China (Li et al. 2014) and 0% in India (Sharma et al. 1998) and Iceland (Haley et al. 2012). Interestingly, a study from other geographic regions of Argentina found that 1.9% of *V. cholerae* non‐O1/non‐O139 carried *stn* (Fraga et al. 2007), further supporting wide variability in the prevalence of this toxin, even between relatively close regions.

One of the most notable findings of our investigation was the detection of two environmental strains, VC92 and VC95, both harbouring *bla*_*CARB‐60*_ and the VPI‐1 element. These strains were isolated 15 days apart from different sampling points approximately 20 km away from each other in and around Córdoba city. VC92 was isolated from the Suquía River at Chacra La Merced, a location where the river has already passed through Córdoba city and is a few kilometres eastward. VC95 was identified in the South master channel at the southern border of the city. Interestingly, the waters transported by the ‘South Master Channel’ are indirectly delivered to the Suquía River via the ‘La Cañada stream’, which joins the river near the central area of Córdoba city. Surprisingly, MLST and phylogenetic analysis revealed that both strains are very closely related. They share the same, previously unreported sequence type ST1563 and are clustered together on a single leaf of the phylogenetic tree. Furthermore, their VPI‐1 elements were identical and SNP analysis of their core genomes showed 0 SNPs between them (data not shown), providing compelling evidence of their high genetic identity. The presence of the VPI‐1 element is particularly significant, as it is widely recognised that VPI‐1 can confer pathogenic and epidemic potential to environmental *V. cholerae* strains (Kumar et al. 2020). This potential is primarily attributed to the expression of the TCP pilus, a crucial virulence factor. Even more surprising was the finding of a TVISS large cluster, which encodes the structural proteins of the TVISS apparatus, within the VPI‐1 element in these strains. Plus, another copy of the TVISS large cluster was perfectly aligned with its expected location in *V. cholerae* O1 ‘El Tor’ reference strain N16961. The finding that the VPI‐1‐related TVISS cluster is of subclass i5, distinct from the subclass i1 of the other copy, indicates that these strains harbour two different TVISS large clusters. The subclass i1 represents the ‘canonical’ cluster located in chromosome II, whereas the subclass i5 is likely mobilised in the context of the VPI‐1 element. Interestingly, based on current information found in the SecReT6 database (Zhang et al. 2023), out of TVISS subclasses i1, i2, i3, i4a, i4b and i5, *Vibrio* species only harbour subclasses i1 and i5, being subclass i1 the only one reported for *V. cholerae*. The rarity of this arrangement is supported by DNA BLAST analysis, since it was found in only two other *V. cholerae* strains (2010V‐116 from Haiti and ICDC‐VC702 from China). This dual presence of different TVISS clusters suggests a complex genetic arrangement derived from genetic exchange with other *Vibrio* species, potentially enhancing the virulence and adaptability of these strains. Consistent with this hypothesis, the VC92 and VC95 strains not only carried more virulence genes than the other *bla*_*CARB*_‐positive strains, but also demonstrated higher enterotoxigenicity in rabbit ileal loops, confirming their enhanced virulent potential.

Finally, we explored the potential genetic relationships between the *V. cholerae* strains harbouring *bla*_*CARB*_ within a global context by extracting their core genomes and constructing a phylogenetic tree that included 46 isolates from diverse locations, dates and sources. Notably, apart from VC92 and VC95, which exhibited a very high degree of genetic identity, our findings reveal considerable genetic diversity among the *V. cholerae* non‐O1/non‐O139 strains. This observation aligns with numerous other reports (Bhandari et al. 2023; Schmidt et al. 2023; Haley et al. 2012; Jiang et al. 2000; Bier et al. 2013), underscoring the heterogeneity within these globally distributed free‐living microorganisms.

As previously mentioned, one limitation of our study is that it includes strains isolated from 1991 to 1994. However, it is important to mention that genomic analysis of *V. cholerae* strains bearing *bla*_*CARB*_ isolated from decades ago in relatively undisturbed environments enables future studies with newer strains to infer potential evolutionary trajectories of *bla*_*CARB*_ in the context of more recent, strong anthropogenic pressures.

Our findings highlight intriguing questions about the evolutionary and ecological mechanisms driving the acquisition and persistence of *bla*_*CARB*_ genes in *V. cholerae* in the context of non‐clinical environments. Given that *V. cholerae* are ancient inhabitants of aquatic ecosystems and that some of the strains we studied were isolated from water bodies with minimal anthropogenic antibiotic exposure, it is likely that natural microbial interactions play a significant role in shaping these resistance patterns. A very interesting publication strongly supports this idea, since the authors were able to recover a freeze‐dried culture of a *V. cholerae* non‐O1/non‐O139 strain isolated in 1916 from a British soldier during World War I, which unexpectedly harboured a *bla*_*CARB‐7*_‐like gene conferring non‐susceptibility to ampicillin decades before the discovery of antibiotics (Dorman et al. 2019). In this context, unravelling the ecological dynamics of microbial interactions is crucial for understanding how antibiotic resistance evolves and spreads in natural settings. Such insights will not only enhance our knowledge of environmental reservoirs of resistance but also inform global efforts to combat antimicrobial resistance through a One Health approach.

## 5 Conclusion

This study addresses the genetic diversity, antimicrobial resistance and virulence potential of environmental *V. cholerae* non‐O1/non‐O139 strains isolated from freshwater sources in Córdoba, Argentina. Our findings reveal that ampicillin resistance in these strains is mediated by CARB‐type β‐lactamases, including four novel variants (CARB‐59 to CARB‐62), likely associated with the *V. cholerae* superintegron. Notably, we identified strains with increased virulent potential linked to unique genetic arrangements of the VPI‐1 pathogenicity island, harbouring both the TCP‐pilus and a TVISS type i5 cluster. Overall, the results of this study underscore the capacity of environmental *V. cholerae* non‐O1/non‐O139 strains to acquire genes, likely through genetic exchange with other environmental bacteria, enhancing their genetic diversity, ecological adaptability, antimicrobial resistance and virulence.

## Author Contributions

**Daiana Guevara Núñez:** investigation, writing – original draft, writing – review and editing, software, methodology, data curation, formal analysis, visualization, validation. **Fabrizzio N. Morandini:** investigation, writing – original draft, methodology, validation, visualization, writing – review and editing, software, formal analysis, data curation. **Geehan Suleyman:** writing – review and editing, methodology, data curation, formal analysis, validation, visualization. **Kyle Crooker:** methodology, validation, visualization, writing – review and editing, formal analysis, data curation. **Jagjeet Kaur:** methodology, validation, visualization, writing – review and editing, formal analysis, data curation. **Gina Maki:** methodology, validation, visualization, writing – review and editing, formal analysis, data curation. **José L. Bocco:** writing – review and editing, conceptualization, funding acquisition, investigation, methodology, supervision. **Darío Fernández Do Porto:** investigation, methodology, validation, visualization, writing – review and editing, software, formal analysis, supervision, data curation. **Markus J. Zervos:** funding acquisition, writing – review and editing, supervision, investigation. **Claudia Sola:** conceptualization, investigation, writing – original draft, methodology, visualization, writing – review and editing, validation, formal analysis, supervision. **H. Alex Saka:** conceptualization, investigation, funding acquisition, writing – original draft, methodology, validation, visualization, writing – review and editing, software, formal analysis, project administration, data curation, supervision, resources.

## Conflicts of Interest

The authors declare no conflicts of interest.

## Supporting information


**Figure S1:** TVISS cluster subclass i1 in VC92 and VC95 strains. Alignment a genomic region coding a TVISS large cluster in strains VC92 and VC95, with *V. cholerae* O1 ‘El Tor’ N16961 (accession: GCF_900205735.1; chromosome 2) and *V. cholerae* non‐O1/non‐O139 2010V‐116 strain (accession: GCF_012275105.1; chromosome 2) performed by pyGenomeViz software. TVISS clusters shown were all predicted as type i1 using SecReT6 v3 online tool. DNA identities ≥ 90% are highlighted. Red arrows within squares highlight genes in the TVISS clusters. DNA sizes are indicated in base pairs (bp) and kilobase pairs (Kbp).

**Table S1:** Sources and susceptibility phenotypes^1^ of environmental *V. cholerae* non‐O1/non‐O139 strains included in this study (*n* = 60).

**Table S2:** Primers used for detection of virulence genes.

**Table S3:** QUAST report of de novo genomes assembly quality.

**Table S4:** CheckM assessment of the assemblies against *Vibrio* genus marker database.

**Table S5:** Virulence genes in environmental *V. cholerae* non‐O1/non‐O139 strains included in this study (*n* = 60).

**Table S6:** Summary of *V. cholerae* strains included in the cladogram.

## Data Availability

All experimental data are accessible for review, either in the manuscript, in a public database, or as material uploaded with the manuscript as additional files.
