# Supplementary material for: Genomic Analysis and Virulence Features of Vibrio cholerae Non‐O1/Non‐O139 Harbouring CARB‐Type β‐Lactamases From Freshwater Bodies, Argentina
Source: Environ Microbiol Rep. 2025 Sep 25;17(5):e70181. doi: 10.1111/1758-2229.70181 (PMC12463395; doi:10.1111/1758-2229.70181)
Supplement: Supplementary file 1 — Figure S1: TVISS cluster subclass i1 in VC92 and VC95 strains. Alignment a genomic region coding a TVISS large cluster in strains VC92 and VC95, with V. cholerae O1 ‘El Tor’ N16961 (accession: GCF_900205735.1; chromosome 2) and V. cholerae non‐O1/non‐O139 2010V‐116 strain (accession: GCF_012275105.1; chromosome 2) performed by pyGenomeViz software. TVISS clusters shown were all predicted as type i1 using SecReT6 v3 online tool. DNA identities ≥ 90% are highlighted. Red arrows within squares highlight genes in the TVISS clusters. DNA sizes are indicated in base pairs (bp) and kilobase pairs (Kbp). [file EMI4-17-e70181-s005.docx]

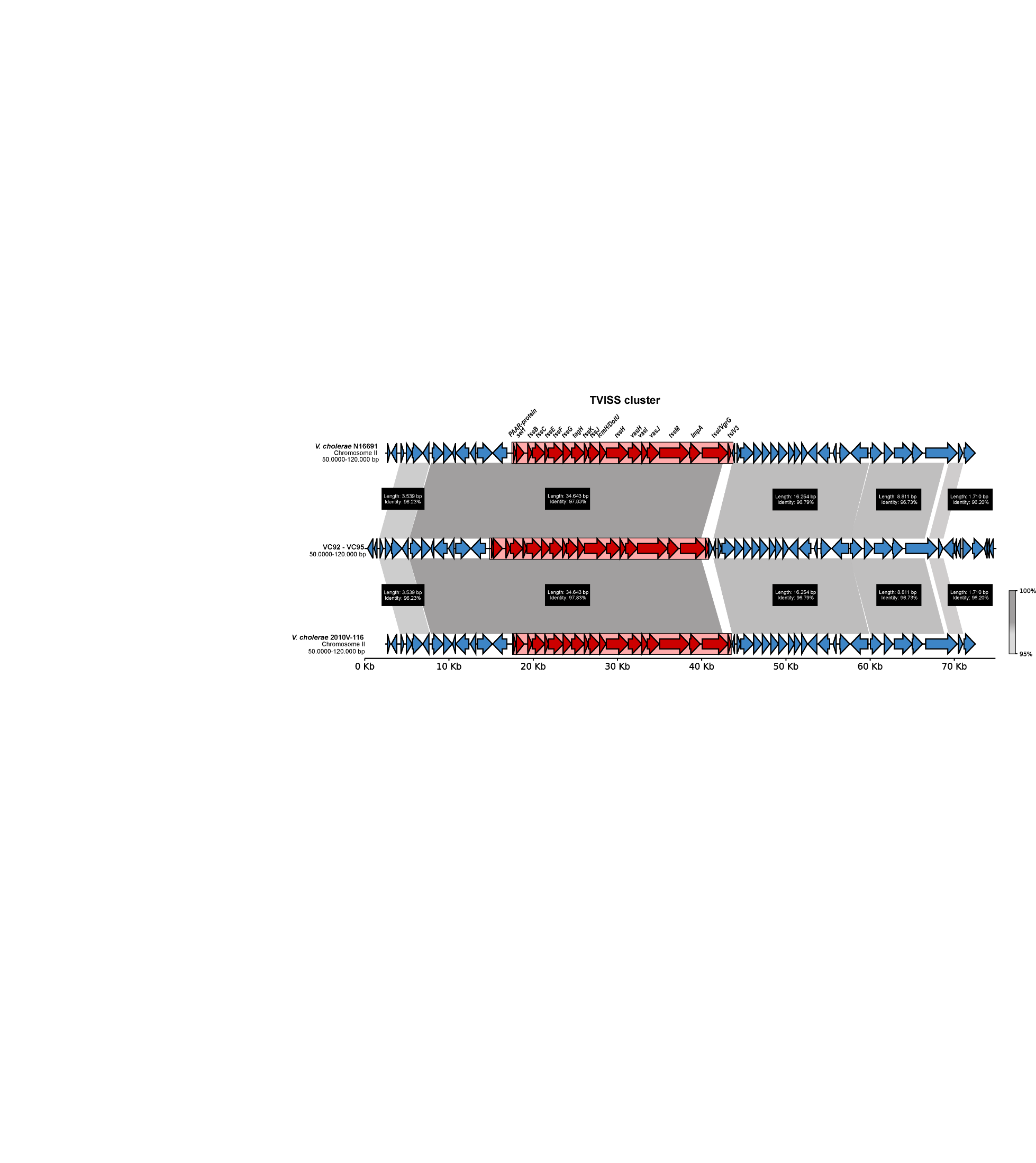


**Figure S1.** **TVISS cluster subclass i1 in VC92 and VC95 strains.** Alignment a genomic region coding a TVISS large cluster in strains VC92 and VC95, with *V. cholerae* O1 “El Tor” N16961 (accession: GCF_900205735.1; chromosome 2) and *V. cholerae* non-O1/non-O139 2010V-116 strain (accession: GCF_012275105.1; chromosome 2) performed by pyGenomeViz software. TVISS clusters shown were all predicted as type i1 using SecReT6 v3 online tool. DNA identities > 90 % are highlighted. Red arrows within squares highlight genes in the TVISS clusters. DNA sizes are indicated in base pairs (bp) and kilobase pairs (Kbp).
