# Supplementary material for: Genomic Analysis and Virulence Features of Vibrio cholerae Non‐O1/Non‐O139 Harbouring CARB‐Type β‐Lactamases From Freshwater Bodies, Argentina
Source: Environ Microbiol Rep. 2025 Sep 25;17(5):e70181. doi: 10.1111/1758-2229.70181 (PMC12463395; doi:10.1111/1758-2229.70181)
Supplement: Supplementary file 2 — Table S1: Sources and susceptibility phenotypes1 of environmental V. cholerae non‐O1/non‐O139 strains included in this study (n = 60). [file EMI4-17-e70181-s007.docx]

**Table S1.**  Sources and susceptibility phenotypes^1^ of environmental *V. cholerae* non-O1/ non-O139 strains included in this study (n=60).

| **Strain ID** | **Isolation date^2^** | **Sampling location** | **Ampicillin susceptibility^3^** |
| --- | --- | --- | --- |
| VC1 | 12/06/1991 | La Cañada stream, Cordoba city, Cordoba, Argentina | Susceptible |
| VC2 | 02/11/1992 | La Cañada stream, Cordoba city, Cordoba, Argentina | Susceptible |
| **VC3** | 02/14/1992 | La Cañada stream, Cordoba city, Cordoba, Argentina | **Resistant** |
| VC6 | 02/27/1992 | Suquia river, Cordoba city, Cordoba, Argentina | Susceptible |
| VC7 | 02/27/1992 | North master channel, Cordoba city, Cordoba, Argentina | Susceptible |
| VC9 | 02/28/1992 | Dolores river, La Cumbre, Cordoba, Argentina | Susceptible |
| VC10 | 02/28/1992 | Calabalumba river, Capilla del Monte, Cordoba, Argentina | Susceptible |
| VC11 | 02/28/1992 | Dolores river, Los Cocos, Cordoba, Argentina | Susceptible |
| **VC12** | 02/28/1992 | San Marcos river, San Marcos Sierras, Cordoba, Argentina | **Resistant** |
| VC13 | 02/28/1992 | Salsacate river, San Carlos Minas, Cordoba, Argentina | Susceptible |
| VC14 | 03/02/1992 | South master channel, Cordoba city, Cordoba, Argentina | Susceptible |
| VC15 | 03/02/1992 | La Cañada stream, Cordoba city, Cordoba, Argentina | Susceptible |
| VC16 | 03/06/1992 | La Cañada stream, Cordoba city, Cordoba, Argentina | Susceptible |
| VC18 | 03/06/1992 | Suquia river, Cordoba city, Cordoba, Argentina | Susceptible |
| VC19 | 03/06/1992 | South master channel, Cordoba city, Cordoba, Argentina | Susceptible |
| VC20 | 03/17/1992 | Suquia river, Cordoba city, Cordoba, Argentina | Susceptible |
| VC21 | 03/17/1992 | North master channel, Cordoba city, Cordoba, Argentina | Susceptible |
| VC22 | 03/17/1992 | South master channel, Cordoba city, Cordoba, Argentina | Susceptible |
| VC23 | 03/17/1992 | La Cañada stream, Cordoba city, Cordoba, Argentina | Susceptible |
| VC24 | 03/17/1992 | South master channel, Cordoba city, Cordoba, Argentina | Susceptible |
| VC25 | 03/17/1992 | Los Sauces river, Nono, Cordoba, Argentina | Susceptible |
| VC28 | 03/23/1992 | South master channel, Cordoba city, Cordoba, Argentina | Susceptible |
| VC29 | 04/10/1992 | South master channel, Cordoba city, Cordoba, Argentina | Susceptible |
| VC30 | 04/10/1992 | Suquia river, Cordoba city, Cordoba, Argentina | Susceptible |
| VC31 | 04/10/1992 | La Cañada stream, Cordoba city, Cordoba, Argentina | Susceptible |
| VC32 | 01/12/1993 | South master channel, Cordoba city, Cordoba, Argentina | Susceptible |
| VC33 | 01/12/1993 | La Cañada stream, Cordoba city, Cordoba, Argentina | Susceptible |
| VC34 | 01/21/1993 | Cosquin river, Cosquin, Cordoba, Argentina | Susceptible |
| VC35 | 01/21/1993 | San Antonio river, Villa Carlos Paz, Cordoba Argentina | Susceptible |
| **VC36** | 01/21/1993 | South master channel, Cordoba city, Cordoba, Argentina | **Resistant** |
| VC37 | 01/21/1993 | La Cañada stream, Cordoba city, Cordoba, Argentina | Susceptible |
| VC38 | 01/21/1993 | Dulce river, Cordoba, Argentina | Susceptible |
| VC39 | 01/28/1993 | La Cañada stream, Cordoba city, Cordoba, Argentina | Susceptible |
| VC40 | 02/05/1993 | North master channel, Cordoba city, Cordoba, Argentina | Susceptible |
| **VC41** | 02/05/1993 | South master channel, Cordoba city, Cordoba, Argentina | **Resistant** |
| VC42 | 02/05/1993 | La Cañada stream, Cordoba city, Cordoba, Argentina | Susceptible |
| VC43 | 02/05/1993 | Suquia river, Cordoba city, Cordoba, Argentina | Susceptible |
| VC46 | 02/10/1993 | Dulce river, Cordoba, Argentina | Susceptible |
| VC50 | 03/01/1993 | Los Molles river, Villla de las Rosas, Cordoba, Argentina | Susceptible |
| VC51 | 03/01/1993 | Los Sauces river, Villa Dolores, Cordoba, Argentina | Susceptible |
| VC52 | 03/01/1993 | Los Sauces river, Villa Dolores, Cordoba, Argentina | Susceptible |
| VC55 | 03/01/1993 | Mina Clavero river, Mina Clavero, Cordoba, Argentina | Susceptible |
| VC57 | 03/09/1993 | La Cañada stream, Cordoba city, Cordoba, Argentina | Susceptible |
| **VC58** | 03/09/1993 | La Cañada stream, Cordoba city, Cordoba, Argentina | **Resistant** |
| VC60 | 03/12/1993 | La Cañada stream, Cordoba city, Cordoba, Argentina | Susceptible |
| VC61 | 03/12/1993 | South master channel, Cordoba city, Cordoba, Argentina | Susceptible |
| VC62 | 03/29/1993 | La Cañada stream, Cordoba city, Cordoba, Argentina | Susceptible |
| VC70 | 10//18/1993 | South master channel, Cordoba city, Cordoba, Argentina | Susceptible |
| VC72 | 10/28/1993 | Suquia river, Cordoba city, Cordoba, Argentina | Susceptible |
| VC73 | 10/28/1993 | South master channel, Cordoba city, Cordoba, Argentina | Susceptible |
| VC76 | 12/02/1993 | Suquia river, Chacra La Merced, Cordoba, Argentina | Susceptible |
| **VC77** | 12/02/1993 | Suquia river, Chacra La Merced, Cordoba, Argentina | **Resistant** |
| VC78 | 12/02/1993 | Suquia river, Chacra La Merced, Cordoba, Argentina | Susceptible |
| VC81 | 12/15/1993 | San Roque dam, Villa Carlos Paz, Cordoba, Argentina | Susceptible |
| VC82 | 12/15/1993 | San Roque dam, Villa Carlos Paz, Cordoba, Argentina | Susceptible |
| VC83 | 12/15/1993 | San Roque dam, Villa Carlos Paz, Cordoba, Argentina | Susceptible |
| **VC84**^4^ | 12/15/1993 | Xanaes river, Arroyito, Cordoba, Argentina | **Resistant** |
| **VC92** | 12/23/1993 | Suquia river, Chacra La Merced, Cordoba, Argentina | **Resistant** |
| **VC95** | 01/07/1994 | South master channel, Cordoba city, Cordoba, Argentina | **Resistant** |
| **VC97** | 11/03/1994 | Anisacate river, Santa Maria, Cordoba, Argentina | **Resistant** |

^1^All strains were phenotypically susceptible to cefazolin, tetracycline, gentamicin, trimethoprim-sulfamethoxazole, chloramphenicol and ciprofloxacin as determined by the disk diffusion method. ^2^Isolation dates are indicated (month/day/year). ^3^Susceptibility phenotypes as determined by the disk diffusion method to ampicillin are shown, with resistant strains highlighted in bold.^4^Later identified as *Vibrio paracholerae* by rMLST analysis.
