# Supplementary material for: Genomic Analysis and Virulence Features of Vibrio cholerae Non‐O1/Non‐O139 Harbouring CARB‐Type β‐Lactamases From Freshwater Bodies, Argentina
Source: Environ Microbiol Rep. 2025 Sep 25;17(5):e70181. doi: 10.1111/1758-2229.70181 (PMC12463395; doi:10.1111/1758-2229.70181)
Supplement: Supplementary file 3 — Table S2: Primers used for detection of virulence genes. [file EMI4-17-e70181-s002.docx]

**Table S2.**  Primers used for detection of virulence genes.

| **Gene** | **Primer** | **Sequence** | **Amplicon** | **Reference** |
| --- | --- | --- | --- | --- |
| *hlyA* | FhlyA | 5´-CTCAGACGGGATTTGTTAGGCACG | 1915 bp | (1) |
|  | RhlyA | 5´-TCTATCTCTGTAGCCCCTATTACG |  |  |
| *toxR* | FtoxR | 5’-CAATGGAATTACCTTGATGTGCAA | 930 bp | (1) |
|  | RtoxR | 5’-GGCTTGAGTCCACCAGTATGTTTT |  |  |
| *ace* | Face | 5’-GCTTATGATGGACACCCTTTA | 284 bp | (2) |
|  | Race | 5’-GTTTAACGCTCGCAGGGCAAA |  |  |
| *zot* | Fzot | 5’-CACTGTTGGTGATGAGCGTTATCG | 243 bp | (2) |
|  | Rzot | 5’-TTTCACTTCTACCCACAGCGCTTG |  |  |
| *ctxA* | FctxA | 5’-CTCAGACGGGATTTGTTAGGCACG | 301 pb | (3) |
|  | RctxA | 5’-TCTATCTCTGTAGCCCCTATTACG |  |  |
| *tcpA* | FtcpA | 5’-GAAGAAGTTTGTAAAAGAAGAACAC | 471 bp | (3) |
|  | RctpA | 5’-GAAAGGACCTTCTTTCACGTTG |  |  |

*V. cholerae* virulence genes correspond to *V. cholerae* cytolysin (*hlyA*), transcriptional activator ToxR/CadC (*toxR*), accessory cholera enterotoxin (*ace*), zonula occludens toxin (*zot*), cholera toxin A subunit (*ctxA*), toxin-coregulated pilus major structural subunit (*tcpA*) and heat-stable enterotoxin NAG-ST (*stn*). Primers for *hlyA* and toxR were designed based on the sequence of *V. cholerae* reference strain N16961 (NCBI accessions: CP028827.1 and CP028828.1). Amplicon sizes are expressed in base pairs (bp).

**References**

1. Bidinost C, Saka HA, Aliendro O, Sola C, Panzetta-Duttari G, Carranza P, Echenique J, Patrito E, Bocco JL. 2004. Virulence factors of non-O1 non-O139 Vibrio cholerae isolated in Cordoba, Argentina. Rev Argent Microbiol 36:158-63.

2. Sharma C, Thungapathra M, Ghosh A, Mukhopadhyay AK, Basu A, Mitra R, Basu I, Bhattacharya SK, Shimada T, Ramamurthy T, Takeda T, Yamasaki S, Takeda Y, Nair GB. 1998. Molecular analysis of non-O1, non-O139 Vibrio cholerae associated with an unusual upsurge in the incidence of cholera-like disease in Calcutta, India. J Clin Microbiol 36:756-63.

3. Heidelberg JF, Eisen JA, Nelson WC, Clayton RA, Gwinn ML, Dodson RJ, Haft DH, Hickey EK, Peterson JD, Umayam L, Gill SR, Nelson KE, Read TD, Tettelin H, Richardson D, Ermolaeva MD, Vamathevan J, Bass S, Qin H, Dragoi I, Sellers P, McDonald L, Utterback T, Fleishmann RD, Nierman WC, White O, Salzberg SL, Smith HO, Colwell RR, Mekalanos JJ, Venter JC, Fraser CM. 2000. DNA sequence of both chromosomes of the cholera pathogen Vibrio cholerae. Nature 406:477-83.
