# Supplementary material for: Genomic Analysis and Virulence Features of Vibrio cholerae Non‐O1/Non‐O139 Harbouring CARB‐Type β‐Lactamases From Freshwater Bodies, Argentina
Source: Environ Microbiol Rep. 2025 Sep 25;17(5):e70181. doi: 10.1111/1758-2229.70181 (PMC12463395; doi:10.1111/1758-2229.70181)
Supplement: Supplementary file 4 — Table S3: QUAST report of de novo genomes assembly quality. [file EMI4-17-e70181-s001.docx]

| Assembly | VC12 | VC36 | VC3 | VC41 | VC58 | VC77 | VC84 | VC92 | VC95 | VC97 |
| --- | --- | --- | --- | --- | --- | --- | --- | --- | --- | --- |
| Mean sequencing depth per contig  (coverage) | 340x | 259x | 280x | 192x | 256x | 150x | 445x | 317x | 122x | 175x |
| Sequencing depth range per genome  (coverage range) | 179x - 2285x | 164x - 1967x | 173x - 2037x | 92x - 1273x | 122x - 1866x | 68x - 1221x | 63x - 8674x | 167x - 2346x | 62x - 975x | 80x - 1102x |
| # contigs  (>= 0 bp) | 160 | 186 | 166 | 187 | 154 | 177 | 172 | 174 | 176 | 87 |
| # contigs  (>= 1000 bp) | 69 | 93 | 75 | 97 | 83 | 92 | 114 | 83 | 86 | 58 |
| # contigs  (>= 5000 bp) | 34 | 48 | 38 | 53 | 40 | 49 | 74 | 50 | 51 | 39 |
| # contigs  (>= 10000 bp) | 32 | 45 | 36 | 46 | 37 | 44 | 70 | 44 | 45 | 33 |
| # contigs  (>= 25000 bp) | 28 | 32 | 30 | 35 | 27 | 36 | 44 | 30 | 31 | 25 |
| # contigs  (>= 50000 bp) | 22 | 23 | 23 | 26 | 22 | 28 | 31 | 21 | 19 | 20 |
| Total length  (>= 0 bp) | 3853235 | 3948853 | 3942239 | 3865022 | 4023302 | 3996764 | 3851034 | 3984080 | 3979613 | 3915275 |
| Total length  (>= 1000 bp) | 3802488 | 3897167 | 3894328 | 3820806 | 3988608 | 3955679 | 3824787 | 3938532 | 3934816 | 3903262 |
| Total length  (>= 5000 bp) | 3742657 | 3812289 | 3826560 | 3737517 | 3885239 | 3868405 | 3742022 | 3867336 | 3860507 | 3854717 |
| Total length  (>= 10000 bp) | 3728134 | 3793363 | 3809996 | 3687427 | 3863915 | 3831465 | 3713309 | 3824830 | 3818001 | 3814870 |
| Total length  (>= 25000 bp) | 3665391 | 3552150 | 3696793 | 3514522 | 3713411 | 3704920 | 3267927 | 3567383 | 3565164 | 3686453 |
| Total length (>= 50000 bp) | 3491327 | 3264683 | 3444810 | 3170284 | 3548125 | 3398819 | 2821332 | 3219766 | 3105954 | 3481705 |
| # contigs | 124 | 148 | 124 | 139 | 114 | 129 | 135 | 125 | 128 | 65 |
| Largest contig | 507544 | 441923 | 407181 | 271862 | 595581 | 307699 | 222754 | 329396 | 359014 | 742500 |
| Total length | 3841252 | 3934956 | 3927821 | 3849820 | 4010118 | 3981467 | 3839211 | 3967155 | 3963271 | 3908090 |
| GC (%) | 47.63 | 47.58 | 47.52 | 47.56 | 47.45 | 47.53 | 47.27 | 47.37 | 47.36 | 47.60 |
| N50 | 188942 | 143803 | 170749 | 119300 | 172440 | 116797 | 71048 | 187614 | 173917 | 200643 |
| N90 | 53226 | 25641 | 39766 | 29873 | 38492 | 41482 | 18628 | 24043 | 24042 | 49895 |
| auN | 203858.5 | 167301.5 | 182193.6 | 127164.4 | 223296.9 | 134074.9 | 87773.5 | 156788.1 | 165972.3 | 275808.3 |
| L50 | 8 | 9 | 8 | 11 | 7 | 11 | 17 | 9 | 9 | 6 |
| L90 | 22 | 32 | 26 | 34 | 24 | 33 | 53 | 31 | 32 | 21 |
| # N's per 100 Kbp | 17.57 | 9.91 | 7.33 | 2.55 | 7.26 | 12.18 | 4.95 | 12.07 | 12.11 | 2.35 |

**Table S3**. QUAST report of *de novo* genomes assembly quality.

Genome coverage is expressed as the “Mean sequencing depth per contig” and the “Sequencing depth range per genome”. GC (%): percentage of G and C nucleotides in the assembly. N50: length for which the collection of all contigs of that length or longer covers at least half an assembly. N90: is the length for which the collection of all contigs of that length or longer covers at least 90% of the assembly. aUN: area under the Nx curve. L50: number of contigs equal to or longer than N50. L90: number of contigs equal to or longer than N90. # N's per 100 Kbp: average number of uncalled bases (N's) per 100,000 assembly bases.
