# Supplementary material for: Genomic Analysis and Virulence Features of Vibrio cholerae Non‐O1/Non‐O139 Harbouring CARB‐Type β‐Lactamases From Freshwater Bodies, Argentina
Source: Environ Microbiol Rep. 2025 Sep 25;17(5):e70181. doi: 10.1111/1758-2229.70181 (PMC12463395; doi:10.1111/1758-2229.70181)
Supplement: Supplementary file 5 — Table S4: CheckM assessment of the assemblies against Vibrio genus marker database. [file EMI4-17-e70181-s006.docx]

**Table S4.** CheckM assessment of the assemblies against *Vibrio* genus marker database.

| Assembly | VC12 | VC3 | VC36 | VC41 | VC58 | VC77 | VC84 | VC92 | VC95 | VC97 |
| --- | --- | --- | --- | --- | --- | --- | --- | --- | --- | --- |
| Marker lineage | Vibrio | Vibrio | Vibrio | Vibrio | Vibrio | Vibrio | Vibrio | Vibrio | Vibrio | Vibrio |
| #Genomes | 70 | 70 | 70 | 70 | 70 | 70 | 70 | 70 | 70 | 70 |
| #Markers | 1084 | 1084 | 1084 | 1084 | 1084 | 1084 | 1084 | 1084 | 1084 | 1084 |
| #Marker sets | 381 | 381 | 381 | 381 | 381 | 381 | 381 | 381 | 381 | 381 |
| 0 | 2 | 1 | 2 | 0 | 0 | 0 | 0 | 0 | 0 | 0 |
| 1 | 1079 | 1079 | 1076 | 1081 | 1083 | 1080 | 1080 | 1080 | 1080 | 1081 |
| 2 | 3 | 4 | 6 | 3 | 1 | 4 | 4 | 3 | 3 | 3 |
| 3 | 0 | 0 | 0 | 0 | 0 | 0 | 0 | 1 | 1 | 0 |
| 4 | 0 | 0 | 0 | 0 | 0 | 0 | 0 | 0 | 0 | 0 |
| 5+ | 0 | 0 | 0 | 0 | 0 | 0 | 0 | 0 | 0 | 0 |
| Completeness | 99.72 | 99.98 | 99.96 | 100.00 | 100.00 | 100.00 | 100.00 | 100.00 | 100.00 | 100.00 |
| Contamination | 0.42 | 0.51 | 0.47 | 0.42 | 0.03 | 0.49 | 0.68 | 0.48 | 0.48 | 0.55 |
| Strain heterogeneity | 0.00 | 0.00 | 0.00 | 0.00 | 0.00 | 0.00 | 0.00 | 0.00 | 0.00 | 0.00 |

Marker lineage: indicates the taxonomic rank of the lineage-specific marker set used to estimated genome completeness, contamination, and strain heterogeneity. #Genomes: number of reference genomes used to infer the lineage-specific marker set. #Markers: number of marker genes within the inferred lineage-specific marker set. #Marker sets: number of co-located marker sets within the inferred lineage-specific marker set. 0-5+: number of times each marker gene is identified. Completeness: estimated completeness of genome as determined from the presence/absence of marker genes and the expected colocalization of these genes. Contamination: estimated contamination of genomes as determined by the presence of multi-copy marker genes and the expected colocalization of these genes. Strain heterogeneity: estimated strain heterogeneity as determined from the number of multi-copy marker pairs which exceed a specified amino acid identity threshold (default = 90%).
