# Supplementary material for: Genomic Analysis and Virulence Features of Vibrio cholerae Non‐O1/Non‐O139 Harbouring CARB‐Type β‐Lactamases From Freshwater Bodies, Argentina
Source: Environ Microbiol Rep. 2025 Sep 25;17(5):e70181. doi: 10.1111/1758-2229.70181 (PMC12463395; doi:10.1111/1758-2229.70181)
Supplement: Supplementary file 6 — Table S5: Virulence genes in environmental V. cholerae non‐O1/non‐O139 strains included in this study (n = 60). [file EMI4-17-e70181-s004.docx]

**Table S5.**  Virulence genes in environmental *V. cholerae* non-O1/ non-O139 strains included in this study (n=60).

| **Strain ID** | **Virulence genes** | | | | | |
| --- | --- | --- | --- | --- | --- | --- |
|  | *hlyA* | *toxR* | *ace* | *zot* | *ctxA* | *tcpA* |
| VC1 | + | + | - | - | - | - |
| VC2 | + | + | - | - | - | - |
| **VC3** | + | + | - | - | - | - |
| VC6 | + | + | - | - | - | - |
| VC7 | + | + | - | - | - | - |
| VC9 | + | + | - | - | - | - |
| VC10 | + | + | - | - | - | - |
| VC11 | + | + | - | - | - | - |
| **VC12** | + | + | - | - | - | - |
| VC13 | + | + | - | - | - | - |
| VC14 | + | + | - | - | - | - |
| VC15 | + | + | - | - | - | - |
| VC16 | + | + | - | - | - | - |
| VC18 | + | + | - | - | - | - |
| VC19 | + | + | - | - | - | - |
| VC20 | + | + | - | - | - | - |
| VC21 | + | + | - | - | - | - |
| VC22 | + | + | - | - | - | - |
| VC23 | + | + | - | - | - | - |
| VC24 | + | + | - | - | - | - |
| VC25 | + | + | - | - | - | - |
| VC28 | + | + | - | - | - | - |
| VC29 | + | + | - | - | - | - |
| VC30 | + | + | - | - | - | - |
| VC31 | + | + | - | - | - | - |
| VC32 | + | + | - | - | - | - |
| VC33 | + | + | - | - | - | - |
| VC34 | + | + | - | - | - | - |
| VC35 | + | + | - | - | - | - |
| **VC36** | + | + | - | - | - | - |
| VC37 | + | + | - | - | - | - |
| VC38 | + | + | - | - | - | - |
| VC39 | + | + | - | - | - | - |
| VC40 | + | + | - | - | - | - |
| **VC41** | + | + | - | - | - | - |
| VC42 | + | + | - | - | - | - |
| VC43 | + | + | - | - | - | - |
| VC46 | + | + | - | - | - | - |
| VC50 | + | + | - | - | - | - |
| VC51 | + | + | - | - | - | - |
| VC52 | + | + | - | - | - | - |
| VC55 | + | + | - | - | - | - |
| VC57 | + | + | - | - | - | - |
| **VC58** | + | + | - | - | - | - |
| VC60 | + | + | - | - | - | - |
| VC61 | + | + | - | - | - | - |
| VC62 | + | + | - | - | - | - |
| VC70 | + | + | - | - | - | - |
| VC72 | + | + | - | - | - | - |
| VC73 | + | + | - | - | - | - |
| VC76 | + | + | - | - | - | - |
| **VC77** | + | + | - | - | - | - |
| VC78 | + | + | - | - | - | - |
| VC81 | + | + | - | - | - | - |
| VC82 | + | + | - | - | - | - |
| VC83 | + | + | - | - | - | - |
| **VC84^1^** | + | + | - | - | - | - |
| **VC92** | + | + | - | - | - | - |
| **VC95** | + | + | - | - | - | - |
| **VC97** | + | + | - | - | - | - |

*V. cholerae* virulence genes detected by PCR correspond to *V. cholerae* cytolysin (*hlyA*), transcriptional activator ToxR/CadC (*toxR*), accessory cholera enterotoxin (*ace*), zonula occludens toxin (*zot*), cholera toxin A subunit (*ctxA*) and toxin-coregulated pilus A subunit (*tcpA*). Ampicillin resistant strains are highlighted in bold.

^1^Later identified as *Vibrio paracholerae* by rMLST analysis carried out upon WGS.
