# Supplementary material for: Genomic Analysis and Virulence Features of Vibrio cholerae Non‐O1/Non‐O139 Harbouring CARB‐Type β‐Lactamases From Freshwater Bodies, Argentina
Source: Environ Microbiol Rep. 2025 Sep 25;17(5):e70181. doi: 10.1111/1758-2229.70181 (PMC12463395; doi:10.1111/1758-2229.70181)
Supplement: Supplementary file 7 — Table S6: Summary of V. cholerae strains included in the cladogram. [file EMI4-17-e70181-s003.docx]

**Table S6.**  Summary of *V. cholerae* strains included in the cladogram.

*Isolates from this study.

| Strain | Year | Location | Serogroup | *bla_CARB_* | Accession | Source |
| --- | --- | --- | --- | --- | --- | --- |
| A186 | 1992 | Argentina | O1 | Negative | GCA_001248135 | Human |
| A200 | 1992 | Argentina | O1 | Negative | GCA_001255295 | Human |
| A201 | 1992 | Argentina | O1 | Negative | GCA_001261515 | Human |
| CCBT0060 | 1992 | Argentina | non-O1/ non-O139 | Negative | ERS2493647 | Environmental |
| CCBT0061 | 1992 | Argentina | non-O1/ non-O139 | Negative | ERS2493648 | Human |
| CCBT0063 | 1992 | Argentina | non-O1/ non-O139 | Negative | ERS2493650 | Human |
| CCBT0101 | 1992 | Argentina | non-O1/ non-O139 | Negative | ERS2493686 | Human |
| CCBT0106 | 1992 | Argentina | non-O1/ non-O139 | Negative | ERS2493691 | Environmental |
| CCBT0112 | 1992 | Argentina | non-O1/ non-O139 | Negative | ERS2493697 | Environmental |
| CCBT0146 | 1992 | Argentina | non-O1/ non-O139 | Negative | ERS2493990 | Environmental |
| CCBT0162 | 1992 | Argentina | non-O1/ non-O139 | Negative | ERS2493715 | Environmental |
| CCBT0242 | 1992 | Argentina | O1 | Negative | ERS2493802 | Human |
| CCBT0271 | 1992 | Argentina | O1 | Negative | ERS2493828 | Human |
| VC001 | 1992 | Argentina | non-O1/ non-O139 | Negative | ERS2708163 | Environmental |
| VC002 | 1992 | Argentina | non-O1/ non-O139 | Negative | ERS2708164 | Environmental |
| VC012 | 1992 | Argentina | non-O1/ non-O139 | Negative | ERS2708174 | Environmental |
| VC12* | 1993 | Argentina | non-O1/ non-O139 | Positive | JBFNBU000000000 | Environmental |
| VC3* | 1992 | Argentina | non-O1/ non-O139 | Positive | JBFNBT000000000 | Environmental |
| VC36* | 1993 | Argentina | non-O1/ non-O139 | Positive | JBFNBV000000000 | Environmental |
| VC41* | 1993 | Argentina | non-O1/ non-O139 | Positive | JBFNBW000000000 | Environmental |
| VC58* | 1993 | Argentina | non-O1/ non-O139 | Positive | JBFNBX000000000 | Environmental |
| VC77* | 1993 | Argentina | non-O1/ non-O139 | Positive | JBFNBY000000000 | Environmental |
| VC92* | 1993 | Argentina | non-O1/ non-O139 | Positive | JBFNCA000000000 | Environmental |
| VC95* | 1994 | Argentina | non-O1/ non-O139 | Positive | JBFNCB000000000 | Environmental |
| VC97* | 1994 | Argentina | non-O1/ non-O139 | Positive | JBFNCC000000000 | Environmental |
| MZO-2 | 2001 | Bangladesh | O14 | Negative | GCF_000153985.2 | Human |
| MZO-3 | 2001 | Bangladesh | O37 | Negative | GCF_000168935.2 | Human |
| TMA 21 | 1982 | Brazil | non-O1/ non-O139 | Negative | GCF_000174295.1 | Environmental |
| 2011EL-1271 | 2011 | Haiti | non-O1/ non-O139 | Positive | GCF_009763665.1 | Human |
| HC-02C1 | 2010 | Haiti | non-O1/ non-O139 | Negative | GCF_000305525.2 | Human |
| HC-41B1 | 2010 | Haiti | non-O1/ non-O139 | Negative | GCF_000304955.2 | Human |
| HE-25 | 2010 | Haiti | non-O1/ non-O139 | Negative | GCF_000279265.1 | Environmental |
| HE-40 | 2010 | Haiti | non-O1/ non-O139 | Negative | GCF_000305115.2 | Environmental |
| HE-45 | 2010 | Haiti | non-O1/ non-O139 | Negative | GCF_000279285.1 | Environmental |
| HE-48 | 2010 | Haiti | non-O1/ non-O139 | Negative | GCF_000220785.1 | Environmental |
| RIMD2214445 | 2001 | Japan | non-O1/ non-O139 | Positive | GCF_023168105.1 | Environmental |
| 1587 | 1994 | Peru | O12 | Negative | GCF_000168895.2 | Human |
| V52 | 1968 | Sudan | O37 | Negative | GCF_000167935.2 | Human |
| VL426 | 2009 | UK | non-O1/ non-O139 | Negative | GCF_000174235.1 | Environmental |
| 2015V-1126 | 2015 | USA | non-O1/ non-O139 | Positive | GCF_009762915.1 | Human |
| BJG-01 | 2010 | USA | non-O1/ non-O139 | Negative | GCF_000221465.1 | Human |
| L6G | 2004 | USA | non-O1/ non-O139 | Positive | GCF_013357685.1 | Environmental |
| PS15 | U | USA | O106 | Negative | GCF_000318075.1 | Environmental |
| RC385AAKH | 1998 | USA | non-O1/ non-O139 | Negative | GCF_000152445.1 | Environmental |
| SL4G | 2004 | USA | non-O1/ non-O139 | Positive | GCF_013357625.1 | Environmental |
| TP | 2011 | USA | non-O1/ non-O139 | Positive | GCF_001857485.1 | Environmental |
